# Improving the Dietary Intake of Health Care Workers through Workplace Dietary Interventions: A Systematic Review and Meta-Analysis

**DOI:** 10.1093/advances/nmab120

**Published:** 2021-11-30

**Authors:** Aasiya Panchbhaya, Christine Baldwin, Rachel Gibson

**Affiliations:** Department of Nutritional Sciences, King's College London, London, United Kingdom; Department of Nutritional Sciences, King's College London, London, United Kingdom; Department of Nutritional Sciences, King's College London, London, United Kingdom

**Keywords:** health care workers, diet, workplace interventions, systematic review, nutritional interventions, occupational nutrition

## Abstract

The workplace has been identified as a potential location for dietary intervention delivery due to the amount of time spent and the meals eaten in this setting. It is recommended that interventions are tailored to specific occupational groups, and to date, there is limited synthesis of the evidence relating to health care workers. This review characterizes and evaluates the effectiveness of dietary interventions in health care workers to aid the design and implementation of interventions. The MEDLINE database was searched to September 2020. The reference list of an umbrella review was hand-searched for additional titles against inclusion criteria. The search included *1*) population, *2*) intervention, and *3*) work environment. Studies were assessed for risk of bias. Harvest plots and forest plots were created to display study quality, direction, and size of effect of selected primary (energy, fruit and vegetable, and fat intake) and secondary outcomes (weight, BMI, blood pressure, and serum cholesterol concentrations). Thirty-nine articles assessing 34 interventions were eligible for inclusion. Intervention types most commonly used were environmental, educational, educational plus behavioral, and behavioral. Due to the heterogeneity in study design and intervention type, results were largely inconclusive. For dietary outcomes, interventions produced small–moderate favorable changes in fruit, vegetable, and fat intake. Decreased fat intake was mainly observed in environmental interventions and increases in fruit and vegetable intake were observed when an educational and/or behavioral component was present. Interventions producing weight loss were mostly nonrandomized trials involving education and physical activity. Total and LDL cholesterol decreased in interventions involving physical activity. Meta-analyses revealed significant decreases in energy intake, weight, blood pressure, total cholesterol, and LDL cholesterol in nonrandomized trials where data were available. Much more research is needed into strategies to promote diet quality improvement in health care workers. A protocol for this review is registered at PROSPERO (CRD42021234906).

## Teaser

**Statement of Significance**: It is recommended that workplace dietary interventions be tailored to specific occupational groups. To our knowledge, this is the first review to examine the effects of dietary workplace interventions in health care professionals. Small–moderate favorable changes in fruit and vegetable intake can be achieved when an educational and/or behavioral component is included in the intervention. For weight loss, interventions involving nutrition education and physical activity in addition to a dietary component show benefit. In the studies reviewed, a high level of heterogeneity was evident and insufficient information reported to ascertain potential bias.

## Introduction

Poor diet quality, excess energy intake, and physical inactivity are primary contributors to the rising prevalence of obesity and consequently to several noncommunicable diseases such as cardiovascular disease, type 2 diabetes, metabolic syndrome, and certain cancers (1). Global obesity prevalence is estimated to increase from 13% to 38% by 2030 if secular trends continue, calling for successful health interventions targeting diet quality and intake (1). Aside from individual health, diet-related illnesses are a huge economic burden, with cardiometabolic diseases costing up to 50.4 billion dollars in the United States (2), and type 2 diabetes alone is estimated to have cost 727 million pounds among 5 European countries in 2015 (3). Therefore, producing effective health-promotion programs is crucial in order to lower the prevalence of diet-related illness, mortality, and health care costs.

One area of particular interest in health promotion research has been the workplace. As the majority of the adult population are part of the workforce (76.4%) and spend one-third of their time in their working environment (4), being at work can be seen as a determinant of one's health. The British Dietetic Association (5) estimates up to 60% of daily food intake occurs in the workplace. An effective workplace health-promotion program therefore has great potential to improve an individual's diet quality—for example, by increasing fruit and vegetable intake and reducing sugar and salt intake. This could also decrease the risk of developing diet-related illness, through weight loss, lowering blood pressure, and serum cholesterol or glucose concentrations.

The present review focuses on workplace well-being intervention research specifically conducted in health care settings, targeting health care employees. Research has shown that obesity rates among health care workers are not significantly different from the general working population. Kyle et al. (6) estimated obesity prevalence among 20,000 health care professionals in England from 2008 to 2012 and found that obesity prevalence was 25% for nurses and 14.4% among “other” health care professionals, which is comparable to other professions. Furthermore, the National Health Interview Survey revealed that the highest prevalence of obesity among occupational groups in the United States included females working in health care support (33.5%) (7). Epidemiological studies of employees with overweight or obesity have identified common characteristics in their working conditions, including long working hours, shift work, and job stress—conditions that are all relevant in health care employees and may make health care workers more susceptible to weight gain (8). Furthermore, absenteeism is highest in health care support occupations in the United States as well as in the UK National Health Service (NHS) (9, 10).

Creating and maintaining a healthy workforce is essential for the performance of health systems. If staff well-being is not maintained, increased absences due to sickness can impact patient care, colleague well-being, and ultimately the health care organization (11). Therefore, research in this area can make a beneficial contribution to policy makers in creating strategies to support health care staff (12) and also build on the WHO's “International Network of Health Promoting Hospitals and Health Services” framework, which emphasizes the need for investing in the safety and wellness of health care employees (13). Previous systematic reviews have evaluated workplace dietary interventions across a range of occupational groups and reported a beneficial effect on dietary outcomes (14). However, as workplaces are highly heterogenous, the application of these findings to specific settings is limited, particularly in health care, where standard working hours are not typical for many health employees.

The aims of this review were as follows: *1*) to characterize dietary interventions tested in health care settings and *2*) to evaluate the effectiveness of these in achieving dietary change in health care employees.

## Methods

A systematic review was conducted following the Preferred Reporting Items for Systematic Reviews and Meta-Analyses (PRISMA) statement (15) and the Cochrane Handbook for Systematic Reviews of Interventions (16). The PICOS (Population, Intervention, Comparison, Outcomes, and Study Design) framework was used to derive the search terms. Studies written in all languages were eligible for inclusion. A protocol for this review is registered at PROSPERO (CRD42021234906).

### Study eligibility criteria

#### Population

Studies including all health care workers working in any health care setting were eligible for inclusion. No restrictions were placed on job role, shift pattern, location, gender, or age in order to gain a full picture of the population working in health care.

#### Intervention

Studies were eligible if they evaluated a dietary intervention or if they were a multicomponent intervention with a dietary element. All workplace interventions were required to have taken place within a health care setting to be eligible. A full list can be found in the search strategy (**Supplemental Table 1**).

#### Comparisons

No restrictions were placed on the comparator and inclusion of a comparator was not required for the study to be included in the present review.

#### Outcomes

During the protocol stage, primary and secondary outcomes were described in general terms as a change in dietary behavior (e.g., food group, nutrient intakes) or nutrition-related health outcomes. These were then refined and focused during the review process according to the most frequently reported outcomes among studies. Outcomes were selected if they were measured in more than 6 studies so that appropriate analysis could be undertaken of these themes.

1. Primary outcomes: Changes in dietary behavior
  - Fruit and vegetable intake
  - Fat intake
  - Energy intake
2. Secondary outcomes: changes in diet-related health outcomes
  - Anthropometry (weight and BMI)
  - Blood pressure (systolic and diastolic)
  - Serum cholesterol concentrations [total, HDL, LDL, and triglycerides (TGs)].

#### Study design

No restrictions were placed on intervention type, and all study designs [randomized controlled trials (RCTs) and nonrandomized controlled trials (NRCTs)] were eligible for review.

### Data sources and search strategy

An umbrella review of 21 systematic reviews investigating the effectiveness of dietary workplace interventions (not specific to health care workers) (14) was hand-searched to identify relevant articles. The related articles identified in PubMed from each review were searched and assessed against the inclusion criteria (snowball search). A search of MEDLINE to identify further relevant studies was undertaken using both free text and Medical Subject Heading (MeSH) terms from 1975 to September 2020. The full search strategy, along with MeSH terms can be found in the supplementary data (Supplemental Table 1). Briefly, the search included *1*) population, *2*) intervention, and *3*) environment.

### Study selection

Articles identified in the search were downloaded into Mendeley (Elsevier) where titles and abstracts were screened for eligibility by a single author (AP). Initially, studies were categorized as “relevant,” “not relevant,” or “unclear.” Full-text screening was performed on articles that were “relevant” and “unclear.” Any studies classified as “unclear” were reviewed by 2 researchers and a consensus reached (RG, CB).

### Risk of bias

Separate tools were used to assess the risk of bias for RCTs and NRCTs. For RCTs, the Cochrane Collaboration's risk-of-bias tool was used (16). Trials were assessed for the following types of bias: selection, performance, detection, attrition, reporting, and “other” bias. Risk of bias for each type was judged as being as “low,” “high,” or “unclear” using standard criteria and a rationale for each judgment was provided.

For NRCTs, the Risk of Bias in Non-Randomized Studies—of Interventions (ROBINS-I) tool was used (17). Initially, the review question and potential confounders were identified. Then, each study was examined for further confounders, and a series of signaling questions were used to enable judgment of multiple types of bias, including selection, misclassification, performance, attrition, detection, and reporting bias (**Supplemental Table 2**). Each type of bias was scored as low, moderate, serious, critical, or unclear using standard criteria (17).

### Data synthesis and analysis

Characteristics of studies were extracted [author, date of publication, outcome(s) measured, participant characteristics, type of intervention, and duration] and tabulated, and this information allowed the grouping of these studies by intervention type, which allowed trends to be identified within multicomponent interventions (**Supplemental Table 3**) (**Table 1**). RCTs and NRCTs were examined for most frequently reported outcomes. Outcome data were further extracted, where available, and study authors were contacted for missing results. Baseline mean, mean change, final values, SDs, and *P* values were tabulated according to primary or secondary outcomes and grouped by study design (**Tables 2** and **3**). Harvest plots were created to display the overall direction of effect as well as additional parameters detailing study design (**Supplemental Figures 1** and **2**). The harvest plot displays interventions as having a significant increase, no effect, or a significant decrease. The height of the bars represents the sample size, shading identifies RCTs, and a lined box signifies whether physical activity measures were used. Numbers within the bars are study identifiers, which can be found in the key (**Supplemental Figure 3**). Exploratory meta-analyses were conducted across all intervention types to gain an overall view of the effect of dietary interventions. The decision to perform meta-analyses was based on assumption of comparability of study population (health care workers) and outcomes (change in specific diet components). For RCTs with complete data, meta-analyses were conducted using Review Manager, which compared workplace intervention with no intervention (RevMan version 5.3; Nordic Cochrane Centre). For NRCTs, the no-intervention group was represented by the baseline measure and the intervention represented by the final value following a period of intervention. Final values as opposed to follow-up values were analyzed as they were more widely available. For studies reporting multiple intervention groups, outcome data were combined using the formula provided in the Cochrane Handbook (16). Random-effects models were used for all analyses to take account of the variability between interventions. A random-effects model assumes heterogeneity of effect across studies. Results were considered statistically significant if *P* < 0.05. Heterogeneity was further assessed by measuring inconsistency (*I*²) and was classified using the Cochrane Handbook as follows: low (0–40%), modest (30–60%), substantial (50–90%), and considerable (75–100%)

**TABLE 1:** Classification of types of dietary interventions used in health care workers

Type of intervention (*n*, number of studies)	Brief description of intervention subtypes (*n*, number of studies)
Educational interventions (*n* = 6)	Face to face nutrition education programs, group meetings (*n* = 4) Internet education (*n* = 1) Education plus financial incentives (*n* = 1)
Environmental interventions (*n* = 8)	Nutrition information through labeling or signage (posters) in workplace cafeterias (*n* = 4) Increased availability of healthier food choices and limited unhealthy food in workplaces (*n* = 1) Choice architecture in workplace cafeterias (*n* = 1) Mixed (*n = 2)* 1+2 (*n =* 1) 1+3 (*n =* 1)
Behavioral interventions (*n* = 7)	Counseling (behavioral/motivational) (*n* = 1) Personalized nutritional status feedback (*n =* 1) Goal setting/intentions (increased healthier food choices) (*n =* 1) Meal replacements (*n* = 1) Behavioral and financial (*n* = 3) Weight-management program/competition plus financial incentives (*n* = 2) Mindful eating training plus price discounts (*n* = 1)
Combined modes of interventions (*n* = 13)	Educational and behavioral (*n* = 8) Education plus counseling/workshops (*n* = 5) Education plus goal setting/planning (*n* = 2) Education plus meetings, goal settings, social support (*n =* 1) Educational and environmental (*n* = 3) Nutrition education plus health campaigns (*n = 1)* Nutrition education, cafeteria changes, plus financial incentives (*n* = 2) Environmental and behavioral (*n* = 2) Increased healthier food choices, group meetings (*n* = 1) Limiting availability of sweet snacks, colleague support, and motivation (*n* = 1)

**TABLE 2:** Summary of outcome data for studies reporting dietary outcomes (energy intake, fruit and vegetable intake, and fat intake)

		Baseline measure (SD), group number (*n*)		Final/follow-up measure (SD), group number (*n*)		
Study (year) (reference)	Outcome measures	Intervention	Control	Intervention	Control	Effect size
Randomized controlled trials						
Energy intake (kcal/d) (kcal/meal)						
Aldana et al. (2005) (45)	Energy intake (kcal/d)	*n* = 662093 kcal/d	*n* = 791793.3 kcal/d	6 wk: *n* = 626 mo: *n* = 616 wk: 1826.2 kcal/d6 mo: 1512.7 kcal/d	6 wk: *n* = 796 mo: *n* = 766 wk:1657.3 kcal/d6 mo:1673.6 kcal/d	6 wk: int: −266.8 kcal vs. −136 kcal in cont (*P* = 0.2435)6 mo: int: −580.3 kcal vs. −119.7 in cont (*P* = 0.0004)
Barratt et al. (1994) (56)	Energy intake (kcal/d)	*n* = not reportedSelf-help: 2077 kcal/dNutrition course: 2388 kcal/daySD not reported	*n* = not reported2197 kcal/dSD not reported	*n* = not reportedSelf-help: 1862 kcal/dNutrition course: 1887 kcal/dSD not reported	*n* = not reported1934 kcal/dSD not reported	Nutrition course vs. self-help (*P* = 0.04)Nutrition course: int. −501 kcal vs. −263 kcal in cont (*P* = 0.05)
Leedo et al. (2017) (24)	Energy intake (kcal/d)	*n* = 591935 (347) kcal/d	*n* = 591935 (347) kcal/d	*n* = 591794 (309) kcal/d	*n* = 591799 (386) kcal/d	Int: −141 kcal vs. −136 kcal in cont (*P* = 0.943)
Lowe et al. (2010) (47)	kcal/meal	*n* = 47Baseline month 1: 665.1 (185.1) kcal/mealBaseline month 2: 572.2 (163.4) kcal/meal	*n* = 49Baseline month 1: 665.1 (185.1) kcal/mealBaseline month 2: 572.2 (163.4) kcal/meal	*n* = 471 mo: 580.4 (159.2) kcal/meal3 mo: 570(179.9) kcal/meal	*n* = 491 mo: 580.4 (159.2) kcal/meal3 mo: 570 (179.9) kcal/meal	No significant results
Stites et al. (2015) (48)	kcal/meal	*n* = 10675.2 kcal/meal (int and cont)SD not reported	*n* = 15675.2 kcal/meal (int and cont)SD not reported	*n* = 10601.1 kcal/mealSD not reported	*n* = 15745.7 kcal/mealSD not reported	Int: −74.1 kcal vs. +70.5 kcal in cont (*P* = 0.01)
Tate et al. (2001) (49)	Energy intake (kcal/d)	*n* = 321558 (654) kcal/d	*n* = 301757 (857) kcal/d	*n* = 323 mo: 1062 (395) kcal/d6 mo: 1146 (450) kcal/d	*n* = 33 mo: 1256 (696) kcal/d6 mo: 1286 (564) kcal/d	3 mo: int: −496 kcal vs. −501 kcal in cont (*P* = NR)6 mo: int: −412 kcal vs. −471 kcal in cont (*P* = 0.88)
Fruit and vegetable intake (servings/d), fruit intake (servings/d), vegetable intake (servings/d)						
Aldana et al. (2005) (45)	Fruit intake (mean servings/d)Vegetable intake (mean servings/d)	*n* = 661.4 servings/d3.2 servings/dSD not reported	*n* = 791.5 servings/d3.3 servings/dSD not reported	6 wk: *n* = 626 mo: *n* = 616 wk: 2.7servings/d6 mo: 2 servings/d6 wk: 4.8 servings/d6 mo: 4.7 servings/dSD not reported	6 wk: *n* = 796 mo: *n* = 766 wk:1.6 servings/day6 mo:1.7 servings/day6 wk: 3.3 servings/day6 mo: 3.4servings/daySD not reported	6 wk: int: +1.3 fruit servings vs. +0.1 in cont (*P* < 0.0001)6 mo: int: +0.6 vs. +0.2 in cont (*P* < 0.0001)6 wk: int: +1.6 veg servings vs. no change in cont (*P* < 0.0001)6 mo: int: +1.5 vs. +0.1 in cont (*P* = 0.0002)
Brug et al. (1999) (55)	Fruit intake (mean servings/d) Vegetable intake (mean servings/d)	*n* = 1521.62 servings/d1.14 servings/dSD not reported	*n* = 1631.61 servings/d1.04 servings/dSD not reported	*n* = 1522.02 servings/d1.07 servings/dSD not reported	*n* = 1631.91 servings/d1.13 servings/dSD not reported	Int: +0.4 vs. +0.3 in cont (NS)Int: −0.07 vs. +0.09 in cont (*P* = <0.07)
Lusczynska and Haynes(2009) (54)	Fruit and vegetable intake (mean servings/d)	*n* = 1042.15 (0.99) servings/d (int and cont)	*n* = 782.15 (0.99) servings/d (int and cont)	*n* = 104 (ITT)2.65 (0.99) servings/d	*n* = 78 (ITT)2.41 (0.84) servings/d	Int: +0.5 servings vs. +0.26 servings in cont (*P* = NR)Cohen's *d* = 0.5 (medium effect size)
Sorensen (1998) (1999) andHunt et al. (2001) (20–22)	Fruit and vegetable intake (mean servings/d)	*n* = 1359Worksite plus family: 2.66 servings/dWorksite: 2.73 servings/dSD not reported	*n* = 13592.66 servings/dSD not reported	*n* = NRWorksite plus family: 2.96 servings/dWorksite: 2.81 servings/dSD not reported	*n* = NR2.62 servings/dSD not reported	Worksite plus family:+16% (equates to 0.4–0.5 servings) vs. −2% in cont (*P* < 0.05)Worksite: +3% (approximately 0.1 servings) vs. −2% cont (*P* > 0.05)
Fat intake (% of EI/d), (g/d), saturated fat intake (g/d)						
Aldana et al. (2005) (45)	Fat intake (% EI/d)Fat intake (g/d)Saturated fat intake (g/d)	*n* = 6634.5% of EI83.5 g/d25 g/dSD not reported	*n* = 7934.3% of EI71.1 g/d20.5 g/dSD not reported	6 wk: *n* = 626 mo: *n* = 616 wk: 27.5% of EI6 mo: 27.8% of EI6 wk: 60.4 g/d6 mo: 48.9 g/d6 wk: 16.6 g/d6 mo: 12.9 g/dSD not reported	6 wk: *n* = 796 mo: *n* = 766 wk: 33.2% of EI6 mo: 35.6% of EI6 wk: 63.2 g/d6 mo: 68.5 g/d6 wk: 18.4 g/d6 mo: 19.7 g/dSD not reported	6 wk: int: −7% EI vs. −1.1% in cont (*P* < 0.0001)6 mo: int: −6.7% vs. +1.3% in cont (*P* < 0.0001)6 wk: int: −23.1 g fat vs. −7.9 g in cont (*P* = 0.0107)6 mo: int: −34.6 g vs. −2.6 g in cont (*P* < 0.0001)6 wk: int.: −8.4 g sat fat vs. −2.4 g in cont (*P* = 0.0009).6 mo: int: −12.1 g vs. −0.8 g in cont (*P* < 0.0001)
Armitage and Conner (2001)(52)	Fat intake (% of EI/d)Fat intake (g/d)Saturated fat intake (g/d)	*n* = 27234.3 (6.432) % EI65.67 (29.19) g/d24.6 (0.69) g/d	*n* = 24435.1 (5.623) % EI64.47 (25.461) g/d24.53 (0.68) g/d	*n* = 27234.6 (6.596) % EI60.02 (25.893) g/d22.32 (0.6) g/d	*n* = 24435.58 (5.779) % EI64.34 (24.367) g/d24.10 (0.64) g/d	*P* (between groups) NRInt: +0.6% EI (*P* = 0.32) vs. +0.48% in cont (*P* = 0.17).Int: −5.65 g fat (*P* < 0.001) vs. −0.13% in cont (*P* = 0.91)Int: −2.28 g sat fat (*P* < 0.001) vs. −0.43 g in cont (*P* = 0.47)
Barratt et al. (1994) (56)	Fat intake (% of EI/d)	*n* = not reportedSelf-help: 37.3% EINutrition course: 37.5% EISD not reported	*n* = not reported36.8% EISD not reported	*n* = not reported.Self-help: 34.9% EINutrition course: 35% EISD not reported	*n* = not reported.34% EISD not reported	Self-help: −2.4% vs. −2% in cont (NS)Nutrition course: −2.5% vs. −2% in cont (NS)
Leedo et al. (2017) (24)	Fat intake (% of EI/d)	*n* = 5930.4 (4.9) %	*n* = 5930.4 (4.9) %	*n* = 5928.6 (4.7) %	*n* = 5930.4 (5.1) %	Int: −1.8% vs. 0% change in cont (*P* = 0.03)
Nonrandomized controlled trials: quasi-experimental						
Lassen et al. (2014) (25)	EI (kcal/meal)Fat intake (% of EI/meal)Fruit and vegetable intake (g/100 g)	*n* = 270 (total)549.3 (500.4) kcal/meal40.4 (28.9) % EI31 (54.4) g/100 g	*n* = 270 (total)501.5 (400.3) kcal/meal43.1 (26.8) %EI24 (37.7) g/100 g	*n* = 270 (total)6 wk: 382.1 (300.2) kcal/meal6 mo: 477.6 (400.3) kcal/meal6 wk: 20.4 (30.1) %EI6 mo: 23.6 (28.5) % EI6 wk: 46 (79.6) g/100 g6 mo: 48 (79.6) g/100 g	*n* = 270 (total)6 wk: 501.5 (400.3) kcal/meal6 mo: 621 (600.7) kcal/meal6 wk: 36.4 (39.3) % EI6 mo: 48.4 (28.1) % EI6 wk: 28 (46.1) g/100 g6 mo: 29 (46.1) g/100 g	6 wk: int: −167.2 kcal vs. −0 kcal in cont (*P* = 0.002)6 mo: int: −71.7 kcal vs. +119.5 kcal in cont (*P* = 0.001)6 wk: int: −20% EI vs. −6.7% in cont (*P* < 0.001)6 mo: int: −16.8% vs. +5.3% in cont (*P* < 0.001)6 wk: int: +15 g vs. +4 g in cont (*P* < 0.001)6 mo: int: +18 g vs. +5 g in cont (*P* < 0.001)
Nonrandomized controlled trials: cross-sectional comparison						
Geaney et al. (2011) (31)	EI (kcal/d)Total fat (g/d)Saturated fat (g/d)	NR	NR	*n* = 501628.6 (406.3) kcal/dTotal fat: 60.5 (22.5) g/dSaturated fat: 20.7 (10.1) g/d	*n* = 501900 (450.8) kcal/dTotal fat: 83.9 (30.4) g/dSaturated fat: 31.9 (14) g/d	Int consumed 298.7 kcal less than cont (*P* = 0.001) Int: consumed 23.4 g less total fat per day than cont (*P* = 0.000).Int: consumed 11.2 g less saturated fat than cont (*P* = 0.000)
Nonrandomized controlled trials: mixed measures						
Armitage (2015) (32)	Portions of fruit/d	Self-generated:*n* = 231.31 (0.33) portions/dVolitional help-sheet:*n* = 331.36 (0.39) portions/d	*n* = 231.3 (0.26) portions/d	Self-generated:*n* = 23 (ITT)1.38 (0.26) portions/dVolitional help-sheet:*n* = 33 (ITT)1.46 (0.27) portions/d	*n* = 23 (ITT)1.25 (0.19) portions/d	Self-generated: +0.07 portions vs. −0.05 portions in cont (*P*: NR)Volitional: +1 portion vs. −0.05 in cont (*P* = 0.001)Self-generated: +0.07 vs. +1 in volitional (*P* = 0.25)

		Baseline measures (SD) and number (*n*)		Final/Follow up measures (SD) and number (*n*)		
Study (year) (reference)	Outcome measures					Effect size
Nonrandomized controlled trials: pre- and post-test						
Energy intake (kcal/d)(kcal/meal)						
Milich et al. (1976) (41)	EI (kcal/meal)	*n* = 470507 kcal/meal		*n* = 4701 wk: 525 kcal/meal2 wk: 459 kcal/meal		1 wk: +18 kcal (*P* > 0.05)2 wk: −48 kcal (*P* < 0.008)
Torquati et al. (2018) (34)	EI (kcal/d)	*n* = 471800 (858) kcal/d		3 mo: *n* = 271842 (861) kcal/d6 mo: *n* = 121683 (569) kcal/d		0–3 mo: +42 kcal, *P* = 0.453–6 mo: −159 kcal, *P* = 0.21
Fruit and vegetable intake						
Blake et al. (2013) (35)	% Eating >5 portions of fruits and vegetables/d	*n* = 145256.9%SD not reported		*n* = 113461%SD not reported		NS
Dawson et al. (2006) (37)	% Reported eating more fruit% Reported eating more vegetables	NR		Eating more fruit: *n* = 188, 29%Eating more vegetables: *n* = 175, 17%		N/A
Hasson et al. (2018), Polaket al. (2015) (38, 39)	Daily consumption of fruits and vegetables, milk products, grains, and protein-rich foods; scale of 1 (never) to 5 (always)	*n* = 1043.17 (0.17)		*n* = 1043.85 (0.15)		+0.68 from baseline *P* < 0.001
Torquati et al. (2018) (34)	Fruit and vegetable intake (% of EI/d)	*n* = 4715.5 (8.2) % EI		3 mo: *n* = 2719.6 (7.8) % EI6 mo: *n* = 1217.7 (9) % EI		0–3 mo: +4.1% EI *P* = 0.453–6 mo: −1.9% EI *P* = 0.21
Fat intake						
Townsend et al. (2016) (50)	Fat in diet score (an overall score of above or equal to 2.5 indicates fat comprises >30% of EI)	Health centers: *n* = 64, 2.77 (0.35)Health systems: *n* = 31, 2.85 (0.45)		Health centers: *n* = 64, 2.56 (0.27)Health systems: *n* = 31, 2.64 (0.36)		Health centers: −0.21 from baseline. *P* not reported.Health systems: −0.21 from baseline. *P* not reported

Cont, control; EI, energy intake; Int, intervention; ITT, intention to treat ; N/A, not available; NR, not reported; NS, not significant (*P* ≥ 0.05).

**TABLE 3:** Summary of outcome data for studies reporting health outcomes (weight, BMI, blood pressure, and cholesterol)

		Baseline measure (SD), group number (*n*)		Final/follow-up measure (SD), group number (*n*)		
Study (year) (reference)	Outcome measures	Intervention	Control	Intervention	Control	Effect size
Randomized controlled trials						
Weight (kg)						
Aldana et al. (2005) (45)	Weight (kg)	*n* = 6689.3 kgSD not reported	*n* = 7985.9kgSD not reported	6 wk: *n* = 626 mo: *n* = 616 wk: 86.4 kg6 mo: 84.9 kgSD not reported	6 wk: *n* = 796 mo: *n* = 766 wk: 85.5kg6 mo: 84.9kgSD not reported	6 wk: int: −2.9 kg vs. −0.4 kg in cont (*P* < 0.0001) 6 mo: int: −4.4 kg vs. −1 kg in cont (*P* < 0.0001)
Choy et al. (2017) (57)	Weight (kg)	*n* = 2071.89 (11.57) kg	*n* = 2271.19 (11.31) kg	*n* = 2070.91 (11.86) kg	*n* = 2270.88 (11.45) kg	Int: −0.98 kg vs. −0.3 kg in cont (*P* not reported)
Leedo et al. (2017) (24)	Weight (kg)	*n* = 5970.4 (10.6) kg	*n* = 5970.4 (10.6) kg	*n* = 5970.3 (10.8) kg	*n* = 5970.3 (11) kg	Int: −0.1 kg vs. −0.1 kg in cont (*P* = 0.461)
Lowe et al. (2010) (47)	Weight (kg)	*n* = 4785.5 (16.2) kg	*n* = 4978.7 (21) kg	*n* = 473 mo: 85.9 (16.8) kg6 mo: 86.7 (16.8) kg12 mo: 86.3 (16.9) kg	*n* = 493 mo: 79.1 (20.5) kg6 mo: 79.6 (20.6) kg12 mo: 80.2 (22) kg	3 mo: int.: +0.4 kg vs. +0.4 kg in cont6 mo: int: +1.2 kg vs. +0.9 kg in cont12 mo: int: +0.8 kg vs. +1.5 kg in cont*P* not reported
Racette et al. (2009) (23)	Weight (kg)	*n* = 6892.4 (24.9) kg	*n* = 5584.5 (20.9) kg	*n* = 6891.6 (25.5) kg	*n* = 5585.1 (23.2) kg	Int: −0.8 kg vs. +0.6 kg in cont (*P* = 0.02)
Tate et al. (2009) (49)	Weight (kg)	*n* = 4677.4 (9.4) kg	*n* = 4578.8 (11.6) kg	*n* = 333 mo: 73.4 kg6 mo: 73.3 kgSD not reported	*n* = 323 mo: 77.1 kg6 mo: 77.2 kgSD not reported	3 mo: int: −4 kg vs. −1.7 kg in cont (*P* = 0.001)6 mo: int: −4.1 kg vs. −1.6 kg in cont (*P* = 0.04)
BMI						
Aldana et al. (2005) (45)	BMI (kg/m²)	*n* = 6632.1 kg/m²SD not reported	*n* = 7931. kg/m²SD not reported	6 wk: *n* = 626 mo: *n* = 616 wk: 31 kg/m²6 mo: 30.5 kg/m²SD not reported	6 wk: *n* = 796 mo: *n* = 766 wk: 31.1 kg/m²6 mo: 31.27 kg/m²SD not reported	6 wk: int: −1.1 kg/m^2^ vs. −0.2 in cont (*P* < 0.0001)6 mo: int: −1.6 vs. −0.03 in cont (*P* < 0.0001)
Brug et al. (1999) (55)	BMI (kg/m²)	*n* = 15224.2 kg/m²SD not reported	*n* = 16323.9 kg/m²SD not reported	*n* = 152N/ASD not reported	*n* = 163N/ASD not reported	Not reported
Choy et al. (2017) (57)	BMI (kg/m²)	*n* = 2028.59 (2.78) kg/m²	*n* = 2228.95 (3.67) kg/m²	*n* = 2028.19 (3.01) kg/m²	*n* = 2228.84 (3.84) kg/m²	Int: −0.4 kg/m^2^ vs. −0.11 in cont (*P* not reported)
Cockcroft et al. (1994) (53)	BMI (kg/m²)	*n* = 4024.9 kg/m²SD not reported	*n* = 4324.48 kg/m²SD not reported	*n* = 4024.36 kg/m²SD not reported	*n* = 4324.49 kg/m²SD not reported	Int: −0.54 kg/m² vs. +0.01 in cont (*P* = 0.025)
Gomel et al. (1993) (19)	BMI (kg/m²)	Education: *n* = 82Behavioral counseling (BC): *n* = 124BC + incentives: *n* = 95Education: 25.5 (3.7) kg/m²BC: 25.5 (4) kg/m^2^BC + incentives: 25.7 (3.9) kg/m²	*n* = 13025.2 (3.8) kg/m²	Not reported	Not reported	BMI increased over all assessment conditions (*P* = 0.04)
Leedo et al. (2017) (24)	BMI (kg/m²)	*n* = 5924.1 (3.5) kg/m²	*n* = 5924.1 (3.5) kg/m²	*n* = 5924.1 (3.6) kg/m²	*n* = 5924.07 (3.6) kg/m²	Int: 0 change vs. −0.3 kg/ m² in cont (*P* = 0.96)
Lusczynska and Haynes (2009) (54)	BMI (kg/m²)	*n* = 104 (int and cont)26.07 (4.96) kg/m²	*n* = 78 (int and cont)26.07 (4.96) kg/m²	*n* = 104 (ITT)25.74 (4.33) kg/m²	*n* = 78 (ITT)27.18 (5.51) kg/m²	Int: −0.33 vs. +1.11 in cont
Racette et al. (2009) (23)	BMI (kg/m²)	*n* = 6834.5 (9.7) kg/m²	*n* = 5531.1 (7.2) kg/m²	*n* = 6834.1 (9.8) kg/m²	*n* = 5531.2 (7.9) kg/m²	Int: −0.4 kg/m² vs. +0.1 in cont (*P* = 0.02)
Blood pressure: systolic and diastolic (mmHg)						
Aldana et al. (2005) (45)	Blood pressure (mmHg)SystolicDiastolic	*n* = 66Systolic: 126.5 mmHgDiastolic: 77.6 mmHgSD not reported	*n* = 79Systolic: 124.6 mmHgDiastolic: 75.6 mmHgSD not reported	6 wk: *n* = 626 mo: *n* = 616 wk: systolic: 119.3 mmHg6 wk: diastolic: 72.7 mmHg6 mo: systolic: 120.6 mmHg6 mo: diastolic: 71.1 mmHgSD not reported	6 wk: *n* = 796 mo: *n* = 766 wk: systolic: 119.2 mmHg6 wk: diastolic: 73 mmHg6 mo: systolic: 120.7 mmHg6 mo: diastolic: 71.8 mmHgSD not reported	Systolic, 6 wk: int: −7.2 mmHg vs. −5.4 mmHg in cont (*P* = 0.3028)Diastolic, 6 wk: int: −4.9 mmHg vs. −2.6 mmHg in cont (*P* = 0.0819)Systolic, 6 mo: int.: −5.9 mmHg vs. −3.9 mmHg in cont (*P* = 0.305)Diastolic, 6 mo: int: −6.5 mmHg vs. −3.8 mmHg in cont (*P* = 0.0506)
Gomel et al. (1993) (19)	Blood pressureSystolicDiastolic	Education: *n* = 82Behavioral counseling (BC): *n* = 124BC + incentives: *n* = 95Education: systolic: 127.6 (11.6) mmHgDiastolic: 82.6 (9.3) mmHgBC: systolic: 130.1 (12.6) mmHgDiastolic: 81.1 (11.5) mmHgBC + incentives: systolic: 124.6 (12.5) mmHg Diastolic: 81.5 (11.1) mmHg	*n* = 130Systolic: 126.1 (12.3) mmHgDiastolic: 96.5 (9.4) mmHg	Not reported	Not reported	Short-term decrease followed by an increase in mean blood pressure for BC + incentives vs. BC (*P* = 0.01)12 mo: decline in mean blood pressure for BC vs. BC + incentives (*P* = 0.0002)
Racette et al. (2009) (23)	Blood pressure (mmHg)SystolicDiastolic	*n* = 68Systolic: 127 (11) mmHgDiastolic: 84 (11) mmHg	*n* = 55Systolic:121 (15)Diastolic: 79 (10)	*n* = 68Systolic: 121(16)Diastolic: 77 (9)	*n* = 55Systolic: 116 (18)Diastolic: 75 (11)	Systolic: int: −6 mmHg vs. −5 mmHg in cont (*P* < 0.01 across sites)Diastolic: −7 mmHg vs. −4 mmHg in cont (*P* < 0.01 across sites)
Serum cholesterol (total, HDL, LDL, TG) (mg/dL)						
Aldana et al. (2005) (45)	Total cholesterol (mg/dL)HDL (mg/dL)LDL (mg/dL)TG (mg/dL)	*n* = 66199.6 mg/dL45.8 mg/dL128.4 mg/dL126.3 mg/dLSD not reported	*n* = 79185.8 mg/dL45.2 mg/dL120.4 mg/dL100.7 mg/dLSD not reported	6 wk: *n* = 626 mo: *n* = 616 wk: 183.6 mg/dL6 mo: 200.4 mg/dL6 wk: 42.7 mg/dL6 mo: 46.1 mg/dL6 wk: 116.1 mg/dL6 mo: 131 mg/dL6 wk: 123.1 mg/dL6 mo: 116.7 mg/dLSD not reported	6 wk: *n* = 796 mo: *n* = 766 wk: 196.2 mg/dL6 mo: 199.5 mg/dL6 wk: 49.4 mg/dL6 mo: 49.6 mg/dL6 wk: 127.6 mg/dL6 mo: 130.1 mg/dL6 wk: 96 mg/dL6 mo: 99.5 mg/dLSD not reported	Total:6 wk: int: −16 mg/dL vs. +10.4 mg/dL in cont (*P* < 0.0001)6 mo: int: +0.8 mg/dL vs. +13.7 mg/dL in cont (*P* = 0. 0153)HDL6 wk: int: −3.1 mg/dL vs. +4.2 mg/dL in cont (*P* = 0.0001)6 mo: int: +0.3 vs +4.4 mg/dL in cont (*P* = 0.0006)LDL6 wk: int: −12.3 mg/dL vs. +7.2 mg/dL in cont (*P* = <0.0001)6 mo: int: +2.6 mg/dL vs. +9.7 mg/dL in cont (*P* = 0.1237)TGs6 wk: int: −3.2 mg/dL vs. −4.7 mg/dL in cont (*P* = 0.8599)6 mo: int: −9.6 mg/dL vs. −1.2 mg/dL in cont (*P* = 0. 4411)
Barratt et al. (1994) (56)	Total cholesterol (mg/dL)	*n* = 668 (total)Self-help: 231.3 mg/dLNutrition course: 237.8 mg/dLSD not reported	*n* = 668 (total)224.7 mg/dLSD not reported	3 mo: *n* = 4176 mo: *n* = 430Self-help:3 mo: 229.3 mg/dL6 mo: 230.53 mg/dLNutrition course:3 mo: 240.11 mg/dL6 mo: 235.8 mg/dLSD not reported	3 mo: *n* = 4176 mo: *n* = 4303 mo: 222 mg/dL6 mo: 224.3 mg/dLSD not reported	Self-help:3 mo: int: −1.93 mg/dL vs. −2.7 mg/dL in cont6 mo: int: −0.77 mg/dL vs. −3.8 mg/dL in cont Nutrition course:3 mo: int: +2.31 mg/dL vs. −2.7 mg/dL in cont6 mo: int: −1.93 mg/dL vs. −3.8 mg/dL in cont
Choy et al. (2017) (57)	Total cholesterol (mg/dL)HDL (mg/dL)LDL (mg/dL)	*n* = 20197.2 (31.6) mg/dL55.5 (15.05) mg/dL122.7 (29.7) mg/dL	*n* = 22215.8 (38.6) mg/dL50.1 (10.4) mg/dL140.9 (33.6) mg/dL	*n* = 20193 (37.4) mg/dL51.3 (11.96)116.6 (35.5) mg/dL	*n* = 22210 (40.9) mg/dL49.8 (10) mg/dL135.9 (30.9) mg/dL	Total: int.: −4.2 mg/dL vs. −5.8 mg/dL in contHDL: int: −4.2 mg/dL vs. −0.3 mg/dL in contLDL: int: −6.1 mg/dL vs. −5 mg/dL in cont
Gomel et al. (1993) (19)	Total cholesterol (mg/dL)	Education: *n* = 82Behavioral counseling (BC): *n* = 124BC + incentives: *n* = 95Education: 196.9 (30.8) mg/dLBC: 199.3 (42.3) mg/dLBC + incentives: 192.3 (38.5) mg/dL	*n* = 130198.7 (42.3) mg/d	Not reported	Not reported	“No significant change”*P* not reported
Lowe et al. (2010) (47)	Total cholesterol (mg/dL)HDL (mg/dL)LDL (mg/dL)TG (mg/dL)	*n* = 47192.4 (32.4) mg/dL58.4 (16.6) mg/dL115.4 (31.6) mg/dL92.9 (42.2) mg/dL	*n* = 49204.1 (41.8) mg/dL58.7 (19.5) mg/dL124.1 (34.4) mg/dL106.5 (68.1) mg/dL	*n* = 47201.8 (28.9) mg/dL60.9 (16.6) mg/dL121.5 (31.3) mg/dL96.8 (41.9) mg/dL	*n* = 49197.4 (42.3) mg/dL57 (16.9) mg/dL120.1 (37.4) mg/dL101.6 (45) mg/dL	Int.: +9.4 mg/dL vs. −6.7 mg/dL (*P* = 0.05)Int: +2.5 mg/dL vs. −1.7 in cont (*P* = 0.05)Int: +6.1 mg/dL vs. +3 mg/dL in cont (*P* = 0.08)Int: +3.9 mg/dL vs. −4.9 mg/dL in cont (*P* = 0.67)
Racette et al. (2009) (23)	Total cholesterol (mg/dL)HDL (mg/dL)LDL (mg/dL)TG (mg/dL)	*n* = 68200 (32) mg/dL56 (16) mg/dL121 (27) mg/dL116 (62) mg/dL	*n* = 55199 (40) mg/dL54 (17) mg/dL121 (35) mg/dL115 (59) mg/dL	*n* = 68192 (32) mg/dL62 (18) mg/dL106 (26) mg/dL118 (60) mg/dL	*n* = 55195 (36) mg/dL61(18) mg/dL109 (32) mg/dL122 (63) mg/dL	Total: int: −8 mg/dL vs. −4 mg/dL in cont (*P* < 0.01 across sites)HDL: int: +6 mg/dL vs. +7 mg/dL in cont (*P* < 0.01) across sitesLDL: int: −15 mg/dL vs. −12 mg/dL in cont (*P* < 0.01) across sitesInt: +2 mg/dL vs. +7 mg/dL in cont (*P* = 0.29)

		Baseline measure (SD) and number (*n*)		Final/follow-up measure (SD) and number (*n*)		
Study (year) (reference)	Outcome measures					Effect size
Nonrandomized controlled trials: quasi-experimental						
Nepper et al. (2020) (26)	BMI (kg/m²)Weight (kg)Blood pressureSystolicDiastolic(mmHg)Total cholesterolHDLLDLTG(mg/dL)	*n* = 4137.7 (6.7) kg/m²101.69 (18.58) kgSystolic: 122.2 (16) mmHgDiastolic: 76 (8.9) mmHg188.8 (8) mg/dL55.9 (5.4) mg/dL107.4 (7) mg/dL26.2 (8.5) mg/dL		*n* = 4135.1 (6.4) kg/m²95.98 (18.58) kgSystolic: 116.5 (17.2) mmHgDiastolic: 68.7 (9.6) mmHg180.6 (8.1) mg/dL60.6 (5.4) mg/dL95 (7.2) mg/dL117.2 (8.7) mg/dL		−2.6 kg/m² from baseline.*P* < 0.0001 (unadjusted)−5.71 kg from baseline (*P* < 0.0001)Systolic: −5.7 mmHg from baseline (*P* = 0.07, unadjusted; 0.16, adjusted)Diastolic: −7.3 mmHg from baseline (*P* = 0.001, unadjusted; 0.005, adjusted)Total cholesterol:−8.2 mg/dL from baseline*P* = 0.2 (unadjusted), 0.08 (adjusted).HDL:+4.7 mg/dL from baseline*P* = 0.33 (unadjusted), 0.32 (adjusted)LDL:−12.4 mg/dL from baseline*P* = 0.08 (unadjusted), 0.17 (adjusted)TGs:−9 mg/dL from baseline*P* = 0.22 (unadjusted), 0.13 (adjusted)
Nonrandomized controlled trial: longitudinal						
Winick et al. (2002) (51)	Weight (kg)	M: *n* = 18101.82 (17) kgF: *n* = 13282.95 (17.4) kg		M: *n* = 1595.45 (18.45) kgF: *n* = 8178.6 (18) kg		M: −6.37 kg from baselineF: −4.35 kg from baseline*P* values not reported
Nonrandomized controlled trials: pre- and post-test						
Weight (kg)						
Hussain et al. (2018) (36)	Weight (kg)	*n* = 13185.09 (11.85) kg		*n* = 13180.5 (12.25) kg		−4.59 kg from baseline (*P* < 0.001)
Lahiri and Faghri (2012) (40)	Weight (kg)	Incentivized:*n* = 35NRNon-incentivized:*n* = 37NR		Incentivized:*n* = 35NRNon-incentivized:*n* = 37NR		Incentivized: −3.31 kg vs. −0.95 kg in non-incentivized (*P* = 0.01)
Rigsby et al. (2009) (42)	Weight (kg)	Individuals participating as a group: *n* = 4289.40 (25.85) kgIndividuals participating individually: *n* = 3093.07 (22.86) kg		Individuals participating as a group: *n* = 4285.95 (25.76) kgIndividuals participating individually: *n* = 3091.26 (22.68) kg		Group: −3.45 kg from baselineIndividual: −1.81 kg from baseline*P* values not reported “Decreased significantly”
Ross and Wing (2016) (43)	Weight (kg)	*n* = 7586.42 (1.94) kg		3 mo: *n* = 7080.64 (5.01) kg6 mo: *n* = 6781.74 (2.53) kg		3 mo: −5.78 kg from baseline (*P* < 0.001)3–6 mo: +1.1 kg (*P* = 0.009)0–6 mo: −4.68 kg (*P* <0.0001)
Torquati et al. (2018) (34)	Weight (kg)	*n* = 4776.3 (17.3) kg		3 mo: *n* = 276 mo: *n* = 123 mo: 76.2 (17.1) kg6 mo: 70.4 (15.7) kg		3 mo: −0.1 kg from baseline.3–6 mo: −5.8 kg*P* values not reported. Weight not addressed.
Townsend et al. (2018) (50)	Weight (kg)	Health centers:81.09 (14.47) kgHealth systems:88.98 (23.83) kg		Health centers:79.37 (23.2) kgHealth systems:87.14 (23.2) kg		Health centers: −1.72 kg from baseline (*P* = 0.001)Health centers: −1.84 kg from baseline (*P* = 0.001)
BMI						
Hussain et al. (2018) (36)	BMI (kg/m²)	*n* = 13134.56 (4.23) kg/m²		*n* = 13132.86 (4.18) kg/m²		−1.71 kg/m² from baseline (*P* < 0.001)
Rigsby et al. (2009) (42)	BMI (kg/m²)	Individuals participating as a group: *n* = 4232.3 (9.8) kg/m²Individuals participating individually: *n* = 3034.6 (8.4) kg/m²		Individuals participating as a group: *n* = 4230.9 (9.8) kg/m²Individuals participating individually: *n* = 3033.8 (8.3) kg/m²		Group:−1.4 kg/m² from baseline.*P*: NR; “decreased significantly”Individual:−0.8 kg/m² from baseline.*P*: NR; “decreased significantly”
Scapellato et al. (2018) (44)	BMI (kg/m²)	*n* = 16727.1 (4.3) kg/m²		*n* = 16726.9 (4.4) kg/m²		−0.2 kg/m² from baseline*P* = 0.03
Torquati et al. (2018) (34)	BMI (kg/m²)	*n* = 4728.3 (6.1) kg/m²		3 mo: *n* = 276 mo: *n* = 123mo: 28.2 (6) kg/m²6 mo: 26.1 (5.7) kg/m²		3 mo: −1 kg/ m² from baseline. *P*: NR..3–6 mo: −2.1 kg/m² from baseline. *P*: NR.
Townsend et al. (2018) (50)	BMI (kg/m²)	Health centers: *n* = 6431.38 (5.7) kg/m²Health systems: *n* = 3134.16 (7.61) kg/m²		Health centers: *n* = 6430.72 kg/ m²SD: NRHealth systems: *n* = 3133.45 kg/ m²SD: NR		Health centers: −0.66 kg/m² from baseline (*P* not reported)Health systems:−0.71 kg/ m² from baseline (*P* not reported).*P* values not reported; “*not significant*”
Blood pressure: systolic and diastolic (mmHg)						
Scapellato et al. (2018) (44)	Blood pressureSystolicDiastolic(mmHg)	*n* = 167Systolic: 129.9 (13.4) mmHgDiastolic: 83 (7.6) mmHg		*n* = 167Systolic: 125.4 (13.4) mmHgDiastolic: 80.5 (8.7) mmHg		Systolic: −5.5 mmHg from baseline (*P* < 0.001)Diastolic: −2.5 mmHg from baseline (*P* < 0.001)
Torquati et al. (2018) (34)	Blood pressureSystolicDiastolic(mmHg)	*n* = 47Systolic: 114.9 (15.2) mmHgDiastolic: 78 (10.1) mmHg		3 mo: *n* = 276 mo: *n* = 123 mo: Systolic: 114.4 (14.1) mmHg; diastolic: 76.8 (10.1) mmHg6 mo: Systolic: 112.6 (13.6) mmHg; diastolic: 74.1 (8.1) mmHg		3 mo:Systolic: −0.5 mmHg from baselineDiastolic: −1.2 mmHg from baseline3–6 mo:Systolic: −1.8 mmHgDiastolic: −2.7 mmHg*P* values not reported
Townsend et al. (2018) (50)	Blood pressureSystolicDiastolic(mmHg)	Health centers:Systolic: 122.2 (15.58) mmHgDiastolic: 78.87 (10.29) mmHgHealth systems:Systolic: 126.68 (16.46) mmHgDiastolic: 80.56 (9.45) mmHg		Health centers:Systolic: 123.1 (11.1) mmHgDiastolic: 77.39 (7.37) mmHgHealth systems:Systolic: 121.87 (13.83) mmHgDiastolic: 78.28 (8.18) mmHg		Health centers:Systolic: +0.97 mmHgDiastolic: −1.48 mmHgHealth systems:Systolic: −4.81 mmHgDiastolic: −2.19 mmHg*P* values not reported; “*not significant*”
Serum cholesterol (total, HDL, LDL, TGs) (mg/dL)						
Scapellato et al. (2018) (44)	Total cholesterolHDLLDLTG(mg/dL)	*n* = 167Total cholesterol:216.1 (31.9) mg/dLHDL:58.9 (16) mg/dLLDL:146.1(28.5) mg/dLTGs:113.8 (57.8) mg/dL		*n* = 167Total cholesterol:203.9 (31.4) mg/dLHDL:55.5 (14.5) mg/dLLDL:137.3 (29.6) mg/dLTGs:112.8 (89) mg/dL		Total cholesterol:−12.2 mg/dL from baseline*P* < 0.001HDL:−3.4 mg/dL from baseline*P* < 0.001LDL:−8.8 mg/dL from baseline*P* < 0.001TGs:−1 mg/dL from baseline.*P* = 0.87

Cont, control; EI, energy intake; Int, intervention; ITT, intention to treat; NR, not reported; TG, triglyceride.

## Results

**FIGURE 1:**
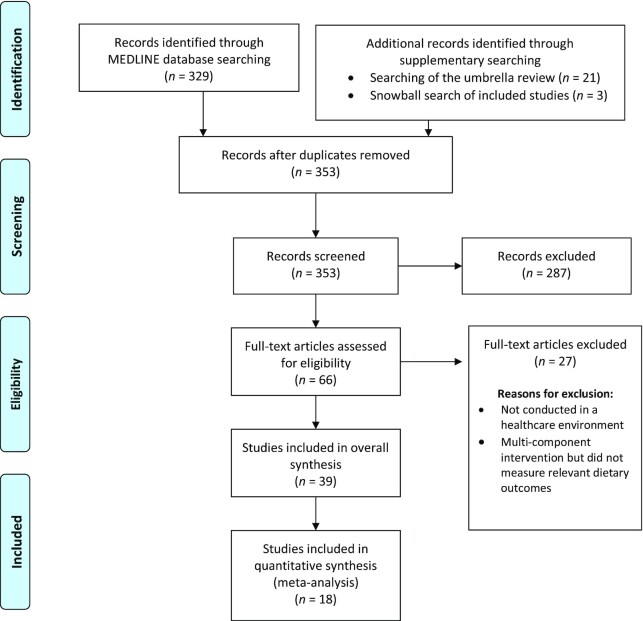
PRISMA flowchart displaying database and supplementary searching and study selection process. PRISMA, Preferred Reporting Items for Systematic Reviews and Meta-Analyses. Reproduced from reference 15.

The MEDLINE search identified 329 potentially relevant articles. An additional 24 records were identified from the umbrella review (14) and snowball search of relevant studies. A total of 287 records were excluded on screening of titles and abstracts. The full text of the remaining 66 articles were reviewed and a further 27 articles were excluded. Thirty-nine articles (representing 34 unique studies) met the inclusion criteria (including 24 identified from the umbrella review and 15 from electronic searching and searching of reference lists). The study selection process is presented in **Figure 1**.

### Characteristics of included studies

A full summary of study characteristics can be found in Supplemental Table 3. All study designs were included, resulting in 16 RCTs (18 articles) and 18 NRCTs (21 articles). From the 16 RCTs identified, 3 were cluster-randomized (18–22), 1 study was cohort-randomized (23), and 1 used a crossover design (24). A range of study designs was observed among NRCTs, including quasi-experimental (25, 26), longitudinal (27–30), cross-sectional comparison (31), mixed measures (32, 33), pilot intervention (34), multilevel ecological (35), and single-arm experimental (36). Eight studies did not state their study design (37–44). The sample sizes of the studies ranged from 26 to 2285 and duration of intervention ranged from 2 wk to 5 y. Multiple outcome measures were reported, with the most common being energy intake, fruit and vegetable intake, fat intake, weight, BMI, blood pressure, and blood lipids.

Sixteen studies were conducted in the United States (18, 20–23, 26, 27–30, 40–43, 45–51), 11 in Europe [United Kingdom (32, 35, 52–54), Ireland (31), Italy (44), Netherlands (33, 55), and Denmark (24)], 3 in Australia (19, 34, 56), 1 in Canada (37), 1 in Israel (38, 39), and 2 in Asia [Hong Kong (57) and Malaysia (36)]. Settings ranged from public and private hospitals, health centers and clinics, nursing homes, and ambulance stations.

### Intervention categories

From the studies identified, 19 interventions exclusively targeted diet, whereas the remaining 15 also targeted changes in physical activity or mood. A variety of intervention types were used and often combined (Table 1). The most common types included environmental, educational plus behavioral element, educational only, and behavioral only. The remaining were a combination of environmental, educational, and behavioral interventions. Educational interventions comprised courses/sessions; environmental interventions were cafeteria changes, events, or campaigns; and behavioral interventions involved counseling or planning.

### Risk of bias

#### Randomized trials

**FIGURE 2:**
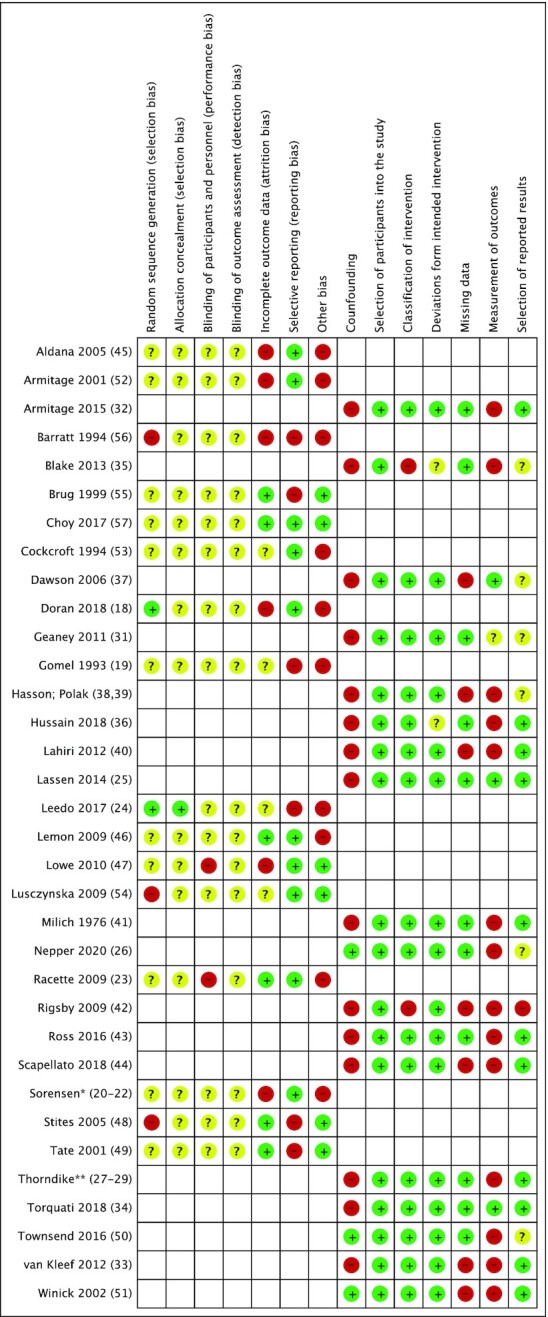
Risk-of-bias summary table for randomized and nonrandomized controlled trials. *Sorensen et al. (20, 21) and Hunt et al. (22); **Thorndike et al. (27), Levy et al. (29), and Dashti et al. (30). +, low risk; −, high risk; ?, unclear risk.

The risk of selection bias in regard to sequence generation was considered high in 3 trials (56, 54, 48), unclear in 11 trials (19–23, 45–49, 52, 53, 55, 57), and low in 2 trials (18, 24) (**Figure 2**). For those considered at high risk, sequence generation included an element of nonrandomization, and for those that were unclear, insufficient information was provided. The risk of selection bias with regard to allocation concealment was considered low in just 1 trial (24); the remaining 15 articles were considered unclear due to insufficient information (18–24, 45–49, 52–57). The risk of performance bias was high in 2 trials (23, 47), which explicitly stated that participants were aware of their intervention status. The remaining 14 were deemed unclear due to authors not addressing this outcome (18–22, 24, 45, 46, 48, 49, 52–57). This was also true for the risk of detection bias; no articles addressed the blinding of outcome assessors and were therefore judged as unclear. The risk of attrition bias was high in 6 trials (18, 20–22, 45, 47, 52, 56), unclear in 4 (19, 24, 53, 54), and low in 6 trials (23, 46, 48, 49, 55, 57). Reasons for high risk included an attrition rate of >20% or incomplete outcome data. Those classified as low risk clearly described rates and reasons for attrition, had a high retention rate, and described the process of adjusting for incomplete outcome data clearly. Reporting bias was considered high in 6 trials due to missing results for outcomes mentioned in the methods (19, 24, 48, 49, 55, 56). The remaining 10 articles reported all of the outcomes that were initially specified (18, 20–23, 45–47, 52–54, 57). Finally, the risk of “other biases” was considered high in 10 trials due to the possibility of inaccurate recall and social desirability bias in self-reported measures and self-selection bias where participants were volunteers (18–24, 45, 46, 52, 53, 56).

#### Nonrandomized trials

For NRCTs, 15 interventions (25, 28–30, 31–44) were at high risk of bias due to confounding as the authors did not control for prognostic variables such as personal motivation and nutritional knowledge; the remaining 3 (26, 50, 51) described appropriate methods to control for these factors. All studies were at low risk of selection bias; participants were not selected based on characteristics observed after the start of the study, and follow-up coincided for most participants. Two studies (33, 41) were judged as at high risk for misclassification; the classification of intervention could have been affected by knowledge of the outcome. Bias due to deviations from intended interventions was considered low risk in 16 studies (25, 28–30, 32–40, 42–44) and unclear in 2 studies (31, 41) where no information was provided. Seven studies (33, 37–40, 42, 44, 51) were judged as at high risk of bias due to missing data either due to attrition rates >20% or incomplete outcome data. Bias in measurement of outcomes was considered high in 14 studies (26, 27–30, 33, 35, 36, 38–44, 50–52); the outcome measure could have been influenced by knowledge of the intervention received. Furthermore, outcome assessors were aware of intervention status. Last, 1 study (42) was judged as at high risk of reporting bias as results from males were excluded due to low sample size.

### Effects on outcome

Of 34 articles, 32 reported a primary or secondary outcome of interest in this review (18–20, 22–25, 27–40, 43, 44, 46–58). Tables 2 and 3 display the results for dietary and health outcomes.

### Primary outcomes

#### Energy intake

**FIGURE 3:**
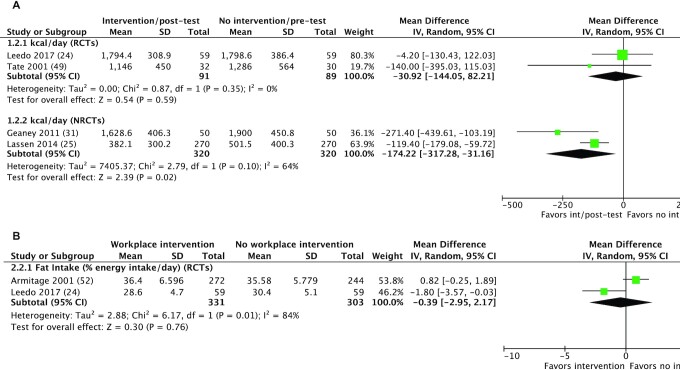
Exploratory meta-analysis of selected dietary outcomes. (A) Meta-analysis of changes in energy intake (kcal/d) in 2 RCTs and 2 NRCTs. RCTs showed no significant differences in energy intake, whereas NRCTs showed a significant decrease in energy intake in intervention groups compared with control groups. (B) Meta-analysis of fat intake (% of energy intake/d) from 2 RCTs showing no significant changes. int, intervention; IV, inverse variance; NRCT, nonrandomized controlled trial; RCT, randomized controlled trial.

Five studies (25, 31, 45, 48, 56) reported a significant decrease in energy intake and the remaining 5 (24, 34, 41, 47, 49) reported no significant changes (Table 2). Those reporting significant effects used a variety of interventions; educational (2/5) (45, 56), environmental (2/5) (25, 48), and behavioral plus financial (1/5) (31). Of the 5 interventions reporting no significant change, 4 used an environmental component (alone or in combination with educational or behavioral strategies) (24, 34, 41, 47). The harvest plot (Supplemental Figure 2) did not reveal trends in sample size for those reporting significant decreases (*n* = 26–683), or for those reporting no effect (*n* = 60–2285). Sixty percent of studies reporting significant effects were RCTs and 1 study used physical activity measures. Similarly, 60% of studies reporting no effect were RCTs and no interventions used physical activity measures. A meta-analysis was performed on 2 of 6 RCTs (24, 41) and 2 of 4 NRCTs (25, 31) (**Figure 3**). The remaining studies were not analyzed due to missing data. Data from RCTs showed no difference in energy intake between groups [mean difference (MD): –30.92 kcal/d; 95% CI: –144.05, 82.21; *P* = 0.59] with no heterogeneity between studies (*I*² = 0%). Data from NRCTs showed that a workplace intervention was associated with a significantly lower energy intake (MD: –174.22 kcal/d; 95% CI: –317.28, –31.16; *P* = 0.02) and moderate heterogeneity (*I*² = 64%).

#### Fruit and vegetable intake

Of the 10 studies measuring fruit and vegetable intake (Table 2), 6 reported significant increases (20–22, 25, 32, 38, 39, 45, 54), 3 reported no significant change (34, 35, 55), and 1 did not report effect size (54). Five of 6 studies producing significant increases in intakes were composed of either a behavioral or educational component (or both) (20–22, 32, 38, 39, 45, 54), with 1 environmental intervention (25). The 3 studies producing no significant change all differed in intervention type: educational (55), educational plus environmental (35), and environmental plus behavioral (34). The harvest plot (Supplemental Figure 2) shows that 5 of 6 studies reporting significant increases involved at least 100 participants, with the remaining study recruiting 79 participants. Fifty percent of studies were RCTs and 50% used physical activity measures. No trends were found in those reporting “no effect” in sample size (*n* = 47–1452) or study design, and 2 of 3 used physical activity measures. A meta-analysis was not performed due to the variety of measuring units between studies.

#### Fat intake

Significant decreases in fat intake were observed in 4 of 7 studies (24, 25, 31, 45) (Table 2). Two of 7 studies reported no difference between groups (52, 56) and the remaining study did not report effect size (51). The studies observing significant decreases were mainly environmental interventions (24, 25, 31), with 1 educational (45). The studies reporting no significant effects were both educational interventions (52, 56). The harvest plot (Supplemental Figure 2) shows no clear trends in sample size (*n* = 60–270), study design (50% RCTs), or use of physical activity measures (25%) for those reporting significant decreases. The 2 studies reporting no effect involved between 6 and 800 participants, were both RCTs, and neither used physical activity measures. Meta-analysis could only be performed on 2 RCTs (24, 52) and showed no differences in fat intake between groups (MD: –0.39%; 95% CI: –2.95%, 2.17%; *P* = 0.76) (Figure 3). Heterogeneity was high (*I*² = 84%). The remaining studies could not be analyzed due to missing data or variance in measurement units.

### Secondary outcomes

#### Weight/BMI

**FIGURE 4:**
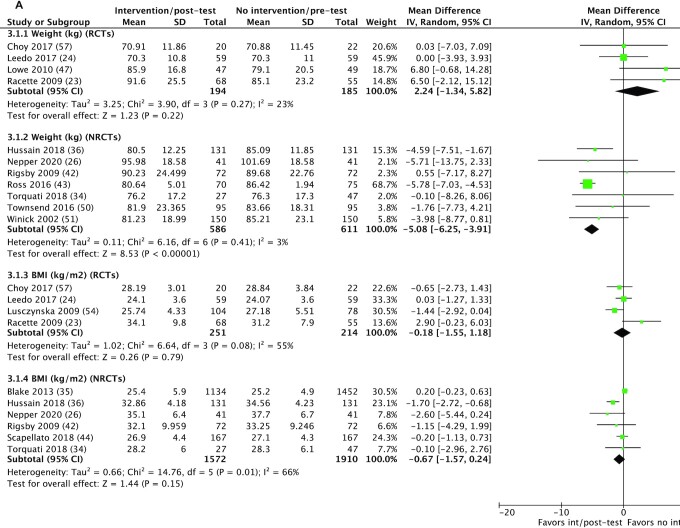

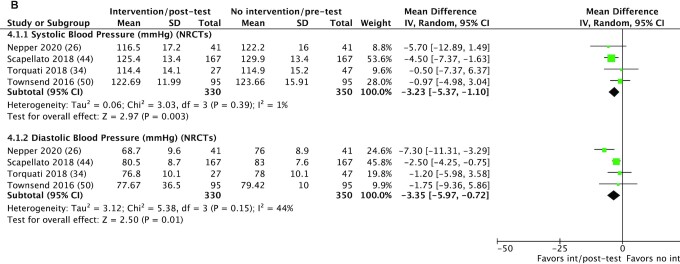
Exploratory meta-analysis of selected weight-related outcomes and blood pressure measurements. (A) 3.1.1–3.1.4. Meta-analysis of selected anthropometric outcomes (weight and BMI) in RCTs and NRCTs. No significant differences were observed in RCTs for weight; however, a significant decrease was observed in NRCTs. No significant differences were observed in BMI in RCTs or NRCTs. (B) 4.1.1 and 4.1.2. Meta-analysis of blood pressure in NRCTs. Significant decreases were observed in systolic and diastolic blood pressure. int, intervention; IV, inverse variance; NRCT, nonrandomized controlled trial; RCT, randomized controlled trial.

Fourteen studies measured changes in weight (23, 24, 26, 36, 40, 42, 43, 45–47, 49–51, 57); 9 interventions reported significant decreases (23, 26, 36, 40, 42, 43, 45, 49–51), 2 reported no significant effects (19, 24, 47), and 3 did not report effect size (but did report a decrease) (46, 51, 57) (Table 3). The harvest plot (Supplemental Figure 3) shows that 8 of 9 interventions reporting a significant decrease involved at least 70 participants, 2 of 3 were NRCTs, and 2 of 3 of interventions used physical activity measures. The 2 interventions reporting no effect involved 60–100 participants, were RCTs, and did not use any physical activity measures. Meta-analysis was conducted on 11 studies (23, 24, 26, 34, 36, 42, 43, 47, 50, 51, 57) (**Figure 4**): 4 RCTs (23, 24, 47, 57) and 7 NRCTs (26, 34, 36, 42, 43, 50, 51). For RCTs, there were no differences in weight between groups (MD: +2.24 kg; 95% CI: –1.34, 5.82 kg; *P* = 0.22) and low heterogeneity (*I*² = 23%). For NRCTs, a significant decrease in weight was observed in the groups receiving the intervention (MD: –5.08 kg; 95% CI: –6.25, –3.91 kg; *P* < 0.001) and low heterogeneity (*I*² = 3%). Studies reporting significant decreases used educational interventions (4 of 9) (26, 36, 45, 50) or an educational component (6 of 9) (26, 36, 43, 45, 49, 50), a behavioral component (5 of 9) (23, 40, 42, 43, 49), environmental component (23) (1 of 9), or a financial incentive (40, 42, 43) (3 of 9). The 2 studies reporting no significant change both comprised an environmental component (24, 47). Of the 10 that were weight-loss interventions (26, 36, 40, 42, 43, 46, 49–51, 57), all reported decreases in weight (not all significant).

BMI was reported in 14 studies (18, 19, 23, 24, 26, 34, 36, 42, 44, 45, 50, 53–55), significant decreases were reported in 7 studies (23, 26, 36, 42, 44, 45, 53), no difference in 4 (24, 34, 54, 50), and a significant increase in 1 study (19) (Table 3). Effect sizes were not reported in 2 studies (18, 55). The harvest plot (Supplemental Figure 3) shows considerable variation in sample size (*n* = 41–297) and study design (43% RCTs) in interventions reporting significant decreases; however, the majority (86%) utilized physical activity measures. Among those displaying no effect, sample size (*n* = 47–182) and study design (50% RCTs) also varied, with 75% utilizing physical activity measures. The RCT reporting a significant increase in BMI had a large sample size (*n* = 431) and used physical activity. Ten studies were entered into meta-analysis (23, 24, 26, 34–36, 42, 44, 54, 57) (Figure 4): 4 RCTs (23, 24, 54, 57) and 6 NRCTs (26, 34–36, 42, 44). Analysis of RCTs and NRCTs showed no differences in BMI (in kg/m²) between groups (MD: –0.18; 95% CI: –1.55, 1.18; *P* = 0.79; moderate heterogeneity: *I*² = 55%; and MD: –0.67; 95% CI: –1.57, 0.24; *P* = 0.15; moderate heterogeneity: *I*² = 66%, respectively). Intervention categories varied for studies reporting significant results; a behavioral component was observed in 5 of 7 studies (23, 36, 42, 44, 53) and an educational component was observed in 4 of 7 studies (26, 36, 45, 53). Similarly, the interventions reporting no significant changes all varied and were a mixture of environmental (24, 34), educational (50, 54), and behavioral strategies (34, 54).

#### Blood pressure

Blood pressure was measured in 7 studies (19, 23, 26, 34, 44, 45, 50) (Table 3). Three interventions reported significant decreases in systolic blood pressure (19, 23, 44, 51). No differences were observed in 3 studies (26, 45, 50), and effect size was not reported in 1 study (34). Four interventions reported significant decreases in diastolic blood pressure (19, 23, 26, 44, 51) and 2 reported no change (45, 50). The harvest plot (Supplemental Figure 3) shows that studies reporting significant decreases in systolic and diastolic blood pressure involved at least 100 participants; 2 of 3 were RCTs and 2 of 3 used physical activity measures. A further study reported a significant decrease in diastolic blood pressure but not systolic; this study had a sample size of 41, was an NRCT, and used physical activity measures (23). The remaining studies reporting no effect displayed no similarities in sample size or study design, but both used physical activity measures (45, 50). Meta-analysis was performed on 4 NRCTs (26, 34, 44, 50) (Figure 4). There was a significant reduction in systolic and diastolic blood pressure in groups receiving the intervention (MD: –3.23 mm Hg; 95% CI: –5.37, –1.1; *P* = 0.003; low heterogeneity: *I*² = 1%; and MD: –3.35 mm Hg; 95% CI: –5.97, –0.72; *P* = 0.01; moderate heterogeneity: *I*² = 44%, respectively). The most common type of interventions producing significant decreases in blood pressure were educational (19, 45, 54) or included a behavioral component (19, 23, 44). All studies reporting no effect were educational strategies (26, 45, 50).

### Serum cholesterol

#### Total cholesterol

**FIGURE 5:**
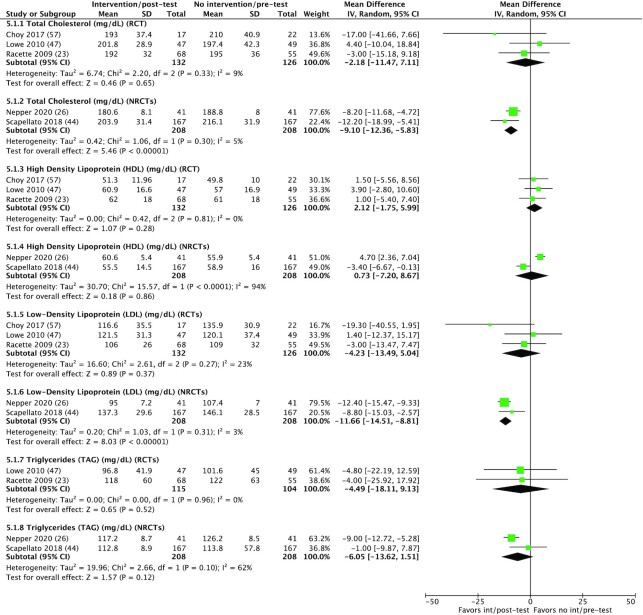
Exploratory meta-analysis of selected serum cholesterol measurements. 5.1.1–5.1.8. Meta-analysis of serum cholesterol concentrations in RCTs and NRCTs (total, HDL, LDL, TGs). No significant differences were observed in total cholesterol for RCTs, whereas total cholesterol significantly decreased in NRCTs. No significant decreases were observed in HDL or LDL in RCTs; however, LDL cholesterol significantly decreased in NRCTs. TGs did not significantly decrease in RCTs or NRCTs. int, intervention; IV, inverse variance; NRCT, nonrandomized controlled trial; RCT, randomized controlled trial; TG, triglyceride.

Total serum cholesterol concentrations were measured in 8 studies (19, 23, 26, 44, 45, 47, 54, 56, 57) and concentrations significantly decreased in 3 studies (23, 44, 45) (Table 3). One study reported a significant increase in total cholesterol (47), no changes were reported in 3 studies (19, 26, 54, 56), and 1 study did not report effect size, although cholesterol concentrations decreased (57). The harvest plot (Supplemental Figure 3) shows that the sample size for those reporting significant decreases was 145 or more, 2 of 3 were RCTs, and all used physical activity measures. For the interventions displaying no effect, no trends were seen in sample size, study design, or use of physical activity. The study reporting a significant increase in total cholesterol used a fairly small sample (*n* = 96). Meta-analysis was performed on 5 studies (23, 26, 44, 47, 57) (**Figure 5**): 3 RCTs (23, 47, 57) and 2 NRCTs (26, 44). For RCTs, there were no differences between groups (MD: –2.18 mg/dL; 95% CI:  –11.47,  7.11; *P* = 0.65), with low heterogeneity (*I*² = 9%). Analysis of NRCTs showed a significant reduction in groups receiving workplace interventions (MD: –9.1 mg/dL; 95% CI: –12.36, –5.83); *P* < 0.001), with low heterogeneity (*I*² = 5%). Interventions that reported the desired effect were educational (1 of 3) (45), behavioral (1 of 3) (44), and environmental and behavioral (1 of 3) interventions (23). The interventions observing no change were educational (37, 54, 56) and the 1 study reporting a significant increase in cholesterol was a combination of environmental, educational, and financial strategies (47).

#### HDL cholesterol

HDL cholesterol was reported for 6 interventions (23, 26, 44, 45, 47, 57) (Table 3). Two studies reported significant increases (23, 47), and the remaining results were mixed with no clear trends emerging. The harvest plot (Supplemental Figure 3) does not reveal any trends in sample size or use of physical activity measures in those reporting significant increases; however, both studies were RCTs. For those reporting no change or decreases, all used physical activity measures (26, 44, 45), A meta-analysis was performed on 5 studies (23, 26, 44, 47, 57) (Figure 5): 3 RCTs (23, 47, 57) and 2 NRCTs (26, 44). There were no differences between groups’ serum HDL cholesterol in the RCTs (MD: 2.12 mg/dL; 95% CI: –1.5, 5.99; *P* = 0.28, with no heterogeneity: *I*² = 0%) and NRCTs (MD: 0.73; 95% CI: –7.2, 8.67; *P* = 0.86, with high heterogeneity: *I*² = 94%). Significant increases in HDL cholesterol were reported in interventions using environmental methods (2 of 2) (23, 47) with one of these also comprising educational and financial components (47). The remaining 4 interventions comprised educational (26, 45) and behavioral interventions (44, 57) (one combined).

#### LDL cholesterol

LDL cholesterol was reported in 6 studies (23, 26, 44, 45, 47, 57); significant decreases were observed in 3 intervention groups (23, 44, 45), no changes were reported in 2 (26, 47), and 1 study did not report effect size but reported a decrease in the intervention group (57) (Table 3). The harvest plot (Supplemental Figure 3) shows that studies reporting significant decreases in LDL cholesterol had a sample size of at least 145 and all used physical activity measures. The 2 interventions displaying no effect observed no similarities in sample size, study design, or use of physical activity measures. A meta-analysis was performed on 5 studies (19, 39, 51, 54, 55) (Figure 5): 3 RCTs (23, 47, 57) and 2 NRCTs (26, 44). No differences were observed in RCTs (MD: –4.23 mg/dL; 95% CI: –13.49, 5.04; *P* = 0.37, with low heterogeneity: *I*² = 23%); however, significant decreases were found in NRCTs (MD: –11.66 mg/dL; 95% CI: –14.51, –8.81); *P* < 0.001, with low heterogeneity: *I*² = 3%). Significant decreases in LDL cholesterol were reported in interventions that were a mixture of intervention types (23, 44, 45). The 2 interventions reporting no change both comprised an educational component, with one also utilizing environmental and financial strategies (47).

#### Triglycerides

No significant changes in TGs were reported in 5 interventions (23, 26, 44, 45, 47) (Table 3). The harvest plot shows no clear trends in sample size or study design; however 4 of 5 interventions utilized physical activity measures (Supplemental Figure 3). Meta-analysis was performed on 4 studies (23, 26, 44, 47) (Figure 5): 2 RCTs (23, 47) and 2 NRCTs (26, 44). There were no differences between groups for RCTs (MD: –4.49 mg/dL; 95% CI: –18.11, 9.13; *P* = 0.52, with no heterogeneity: *I*² = 0%) and NRCTs (MD: –6.05 mg/dL; 95% CI: –13.62, 1.51; *P* = 0.12, with moderate heterogeneity: *I*² = 62%). Two of 5 interventions were educational (26, 45), and the remaining were a mixture of educational, behavioral, and environmental (23, 44, 47).

## Discussion

The purpose of this systematic review was to characterize and evaluate the effectiveness of interventions used in health care settings to improve the dietary intake of health care workers. Thirty-four interventions (from 39 articles) were included, with the majority using 1 strategy or a combination of educational, behavioral, and environmental strategies. Nineteen were solely dietary interventions, whereas 15 were multicomponent. Harvest plots showed that two-thirds of interventions produced favorable changes in fruit, vegetable, and fat intake; however, they did not display a particular trend in sample size, study design, or use of physical activity measures. Harvest plots also revealed that the majority of significant decreases in weight, BMI, and blood lipid measurements were displayed in interventions that utilized physical activity measures. Meta-analyses revealed significant decreases in energy intake, weight, blood pressure, total cholesterol, and LDL cholesterol in NRCTs in groups receiving workplace interventions. However, this is not reflected in the harvest plots, which did not display trends in study design for any dietary or health outcomes except for weight. These differences can be explained by the fewer datasets entered into meta-analysis where data were unavailable. Meta-analysis did not reveal favorable changes in any RCTs, emphasizing the unclear trends observed in the harvest plots.

Overall analysis of outcome data suggested that the most effective interventions comprised an educational component (67%); small favorable trends point towards environmental interventions for decreasing fat intake and combined educational and behavioral interventions for weight-related and health outcomes.

### Dietary outcomes

Fifty percent of studies in this review measuring energy intake reported significant decreases in calories eaten or purchased, and these studies were a mixture of environmental (cafeteria changes), educational, or behavioral/financial interventions. Similarly, more than half of the interventions measuring fruit and vegetable intake reported significant increases, which tended to implement educational and/or behavioral strategies. Previous reviews, however, report similar effect sizes in environmental interventions. Allan et al. (58) evaluated the effectiveness of 22 environmental workplace interventions and found that 50% reported significantly higher fruit and vegetable consumption and reductions in calories purchased. Furthermore, Geaney et al. (59) evaluated the effectiveness of workplace dietary interventions in 6 studies and found that environmental and educational interventions produced small increases in fruit and vegetable intake. Previous reviews tend to be consistent in that favorable changes in energy and fruit and vegetable intake tend to be modest to moderate. However, a systematic review by Hendren and Logomarsino (60) found a moderate to strong association between cafeteria changes and increased fruit and vegetable intake, with 13 of 18 studies reporting favorable increases. Environmental interventions may therefore be a useful direction for further workplace diet quality interventions. However, the authors acknowledge that the results must be interpreted with caution due to the high amount of self-reported measures and heterogeneity between studies. Also, the review was not specific to health care workers and therefore has limited applicability.

Over half of the studies measuring fat intake reported significant decreases, with the majority using environmental strategies (changes in cafeteria menu items). The studies reporting no changes were both educational interventions. Larger effect sizes have been observed in previous reviews (61, 62), which found that the majority of studies they evaluated reported significant decreases in fat intake. Engbers et al. (61) observed this finding in environmental interventions, which is consistent with the findings in this review; however Mhurchu et al. (62) did not analyze fat intake according to intervention type. Both reviews were also not specific to health care workers. Despite this, the common theme of environmental interventions may be valuable in designing future worksite interventions.

### Weight/BMI

Favorable weight outcomes were observed in interventions comprising educational and behavioral components, such as group lessons and behavioral counseling. Over half of the studies observing weight loss were multicomponent, and all except one reporting significant decreases in BMI encouraged physical activity. These findings were also observed by Anderson et al. (8), who reviewed the effectiveness of worksite nutrition and physical activity interventions and reported a modest reduction in weight status, with intervention categories most commonly reported as educational and behavioral. Although this review is not specific to health care workers, Power et al. (63) reviewed 13 RCTs on workplace diet and physical activity interventions in health care professionals and found that weight was significantly reduced in the intervention groups after 12 mo. Therefore, this review is consistent with previous research demonstrating that educational and behavioral strategies combined with physical activity measures produce the most effective weight-loss outcomes. Upadhyaya et al. (64) reviewed 51 worksite obesity interventions in health care workers and found behavioral and educational multicomponent strategies to produce the most significant effects on weight but found inconclusive results when comparing educational and behavioral strategies directly. The majority of interventions reporting favorable weight outcomes had also specified weight loss as a primary outcome, which may, in part, account for the differences between the studies that did not report significant changes. It is also important to consider that physical activity measures may have also contributed to weight loss, so the extent of the dietary component independently causing weight loss cannot be established without individual-level data.

### Diet-related measures of cardiovascular health

More studies reported significant decreases in diastolic blood pressure (66%) than systolic blood pressure (33%), and the most effective interventions tended to be educational or behavioral. In terms of blood lipids, total and LDL cholesterol significantly decreased and HDL cholesterol increased in approximately 50% of studies. No studies reported significant effects on TGs. The most common intervention type was educational, with few using behavioral and financial incentives. This review observed greater effects than previous reviews. A systematic review evaluating workplace interventions measured both blood pressure and blood lipids and found the evidence largely inconclusive (65). This has been reiterated by an additional review (66) that evaluated internet-based worksite interventions and found more nonsignificant effects on blood pressure than significant, and large inconsistencies in results for blood lipids, making conclusions impossible. It may be important to note that these reviews were not specific to health care workers, which may, in part, account for the differences observed.

### Study design

A greater proportion of NRCTs reported favorable outcomes compared with RCTs; however, most NRCTs were at high risk of bias due to confounding, as many prognostic variables were not controlled for, limiting the ability to associate the intervention with outcome. It is possible that the magnitude of effect size was overestimated compared with RCTs due to poor study design (67). However, the quality of the RCTs in this review is also questionable. Although RCTs are considered the gold standard, risk-of-bias assessments revealed that the majority did not provide enough information to judge the risk of selection, performance, or detection bias, and many were at high risk of bias due to self-reporting. Therefore, the results from both the RCTs and NRCTs in this review must be considered with caution.

### Strengths

To our knowledge, this is the first review to examine the effects of dietary workplace interventions in all health care professionals and can therefore make a valuable contribution to this research area. To capture all relevant studies, the database search was carried out without restrictions on health care setting, health care population, study design, language, or date of publication. Using both specific (MEDLINE) and generic search (snowball search) methods allowed the capturing of studies that may have been missed if just 1 search type was used. This has been demonstrated in a study comparing search methods (68), which found that only 7% of studies were identified by both searches and that a generic search identified more relevant studies (51%) than a specific search (41%). The umbrella review (15) also used multiple databases to search for studies, ensuring a wide range of studies were captured. In addition, focusing on a specific work environment allowed specific recommendations to be made to this research area.

### Limitations

Comparisons between all studies were impossible due to study design, variety of outcomes, and missing data. It was not possible to perform a meta-analysis on fruit and vegetable intake due to various reporting methods and lack of standard serving sizes, and for all outcomes, a complete meta-analysis was impossible due to missing data. Authors were contacted in order to overcome this; however, not all data were retrieved. Without participant-level data it is not possible to determine if changes in biomarkers such as blood lipids are independent of weight loss. Multiple intervention types were combined and entered into meta-analysis, which could explain the large heterogeneity found. However, as workplace interventions are multicomponent in nature, it is impossible to measure each intervention type individually. The risks of bias in RCTs were largely unclear due to missing information, and many were at high risk of reporting bias due to selective reporting, social desirability bias, or inaccurate recall. Almost all NRCTs were at high risk of confounding and many failed to control for prognostic variables, limiting the ability to establish causality. Analysis of specific subgroups (job role, shift pattern, ethnicity) was not possible due to limited reporting. An additional limitation is that only 1 database was searched (MEDLINE). However, as the search process included hand-searching the reference list of a recently published umbrella review (14), which used comprehensive search methods, the authors did not deem it necessary to search more than 1 database. A further limitation is that the selection of studies was initially carried out by a single author. However, the initial selection involved extracting studies from a recently completed umbrella review (14), where duplicate screening was utilized.

**Table 4** outlines recommendations for future research, which addresses the issues surrounding study design, reporting methods, and remaining gaps in research. With regard to study design, future research would benefit from controlling variables such as health care subgroups and shift pattern in order for specific and tailored interventions to be designed. Multicomponent interventions may also benefit from controlling for physical activity in order to measure the effectiveness of the dietary component alone as well as both combined. In addition, shortening the recall period time would reduce the risk of self-report and recall bias. To address reporting issues, research should be very transparent within their methodology. For example, randomized trials should explicitly state the details surrounding sequence generation and blinding. Furthermore, studies would benefit from reporting all outcome data to ensure transparency as well as to allow for further review to be undertaken by researchers. Finally, further research is recommended following the results of this review. This includes more research into increasing fruit and vegetable intake as the present review contradicts previous research in terms of intervention type. Also, more workplace interventions focusing on different dietary components, such as sugar and salt intake, would be beneficial as they are key contributors to diet-related illness and were not widely measured in the research found.

**TABLE 4:** Recommendations for future research to determine efficacy of workplace interventions in improving diet and diet-related health outcomes in health care workers

	Recommendations
Study design	Future research may benefit from controlling for health care subgroups and shift patterns to allow for more thorough comparison and therefore the design of effective interventions
	A minimum follow-up time of 12 mo may help establish whether interventions are sustainable
	Future research may benefit from controlling for physical activity in combined interventions to allow the measurement of the effectiveness of dietary and physical elements alone, as well as combined
	To reduce the risk of self-report and recall bias, future research may benefit from shortening the recall period time
Reporting	Randomized trials may benefit from clearly reporting details surrounding sequence generation, concealment and blinding so that the risk of bias can be appropriately assessed
	Explicitly stating the recall period length will allow an appropriate assessment of recall bias
	Reporting all outcome data would allow for a complete meta-analysis to be performed, and therefore the ability to make reliable associations between intervention and effect
Research gap	This review found that educational and/or behavioral strategies were most effective in increasing fruit and vegetable intake, whereas previous reviews have focused on environmental change. Further research into these strategies can clarify the most effective intervention type
	Consistent with previous reviews, this review found that environmental interventions were effective in reducing fat intake; research on specific subgroups and shift patterns can aid the design of tailored interventions
	Outcomes such as sugar and salt intake were not widely measured. As these are key contributors to diet-related illness, it may be beneficial to investigate interventions aiming to reduce sugar and salt intake

### Conclusions

Overall, research into worksite dietary interventions among health care workers seems to be highly heterogenous in terms of study design, sample size, and intervention type. The current paper suggests that, for decreasing fat intake, environmental interventions via cafeteria changes produce the most effective change, and educational and/or behavioral interventions produced increases in fruit and vegetable intake. No specific intervention type was more beneficial with regard to reducing energy intake. Significant weight loss and decreases in total and LDL cholesterol were observed most in interventions that included physical activity parameters. More focused research is needed to identify interventions to improve dietary intake. A favorable trend pointed towards educational and behavioral interventions for weight-related and health outcomes; however, comparison and the ability to make definitive conclusions were difficult due to heterogeneity, missing data, and high or unclear risks of bias in studies.

## Supplementary Material

nmab120_Supplemental_File

## Notes

The authors report no funding received for this work.

Author disclosures: The authors report no conflicts of interest.

Supplemental Figures 1–3 and Supplemental Tables 1–3 are available from the “Supplementary data” link in the online posting of the article and from the same link in the online table of contents at https://academic.oup.com/advances/.

Abbreviations used: MD, mean difference; MeSH, Medical Subject Heading; NRCT, nonrandomized controlled trial; RCT, randomized controlled trial; TG, triglyceride.
