# Supplementary material for: Improving the Dietary Intake of Health Care Workers through Workplace Dietary Interventions: A Systematic Review and Meta-Analysis
Source: Adv Nutr. 2021 Nov 30;13(2):595–620. doi: 10.1093/advances/nmab120 (PMC8970821; doi:10.1093/advances/nmab120)
Supplement: nmab120_Supplemental_File [file nmab120_supplemental_file.docx]

| **Supplementary Table 1: Search Strategy** | | |
| --- | --- | --- |
|  | Searches | Results |
| 1 | exp *health personnel/ or exp *community health workers/ or exp *dental auxiliaries/ or exp *dental assistants/ or exp *dental hygienists/ or exp *dental technicians/ or exp *denturists/ or exp *emergency medical technicians/ or exp *home health aides/ or exp *licensed practical nurses/ or exp *nursing assistants/ or exp *psychiatric aides/ or exp *operating room technicians/ or exp *pharmacy technicians/ or exp *physical therapist assistants/ or exp *physician assistants/ or exp *ophthalmic assistants/ or exp *pediatric assistants/ or exp *anesthetists/ or exp *anesthesiologists/ or exp *nurse anesthetists/ or exp *audiologists/ or exp *dental staff/ or exp *dental staff, hospital/ or exp *dentists/ or exp *dentists, women/ or exp *endodontists/ or exp *"oral and maxillofacial surgeons"/ or exp *orthodontists/ or exp *doulas/ or exp *emergency medical dispatcher/ or exp *faculty, dental/ or exp *faculty, medical/ or exp *faculty, nursing/ or exp *medical staff/ or exp *medical staff, hospital/ or exp *hospitalists/ or exp *nurses/ or exp *nurse practitioners/ or exp *family nurse practitioners/ or exp *pediatric nurse practitioners/ or exp *nurse specialists/ or exp *nurse clinicians/ or exp *nurse midwives/ or exp *nurses, pediatric/ or exp *nurses, neonatal/ or exp *nurses, community health/ or exp *nurses, international/ or exp *nurses, male/ or exp *nurses, public health/ or exp *nursing staff/ or exp *nursing staff, hospital/ or exp *nutritionists/ or exp *occupational therapists/ or exp *optometrists/ or exp *personnel, hospital/ or exp *pharmacists/ or exp *physical therapists/ or exp *physician executives/ or exp *physicians/ or exp *allergists/ or exp *cardiologists/ or exp *dermatologists/ or exp *endocrinologists/ or exp *foreign medical graduates/ or exp *gastroenterologists/ or exp *general practitioners/ or exp *geriatricians/ or exp *nephrologists/ or exp *neurologists/ or exp *occupational health physicians/ or exp *oncologists/ or exp *radiation oncologists/ or exp *ophthalmologists/ or exp *osteopathic physicians/ or exp *otolaryngologists/ or exp *pathologists/ or exp *pediatricians/ or exp *neonatologists/ or exp *physiatrists/ or exp *physicians, family/ or exp *physicians, primary care/ or exp *physicians, women/ or exp *pulmonologists/ or exp *radiologists/ or exp *rheumatologists/ or exp *surgeons/ or exp *barber surgeons/ or exp *neurosurgeons/ or exp *orthopedic surgeons/ or exp *urologists/ or exp *students, health occupations/ or exp *students, dental/ or exp *students, medical/ or exp *students, nursing/ or exp *students, pharmacy/ or exp *students, premedical/ | 425521 |
| 2 | (diet* adj intervention*).mp. | 9044 |
| 3 | (nutrit* adj intervention*).mp. | 7256 |
| 4 | (health* adj intervention*).mp. | 15737 |
| 5 | (work* adj intervention*).mp. | 1852 |
| 6 | (nutrit* adj program*).mp. | 3505 |
| 7 | (health* adj program*).mp. | 48304 |
| 8 | health promotion.mp. or exp *Health Promotion/ | 94800 |
| 9 | (nutrit* adj education*).mp. | 5709 |
| 10 | (diet* adj education*).mp. | 737 |
| 11 | (nutrit* adj counselling).mp. | 489 |
| 12 | (diet* adj advice*).mp. | 2395 |
| 13 | 2 or 3 or 4 or 5 or 6 or 7 or 8 or 9 or 10 or 11 or 12 | 180030 |
| 14 | 1 and 13 | 8589 |
| 15 | 14 not patient*.mp. | 5651 |
| 16 | exp *Workplace/ or workplace.mp. | 51054 |
| 17 | worksite.mp. | 2931 |
| 18 | (work* adj environment*).mp. | 16190 |
| 19 | (work* adj setting*).mp. | 2367 |
| 20 | 16 or 17 or 18 or 19 | 64564 |
| 21 | 1 and 13 and 20 | 472 |
| 22 | 21 not patient*.mp. | 336 |
| 23 | limit 23 to humans | 329 |

**Supplemental Table 2: ROBINS-I tool for assessing risk of bias in non-randomised controlled studies.**


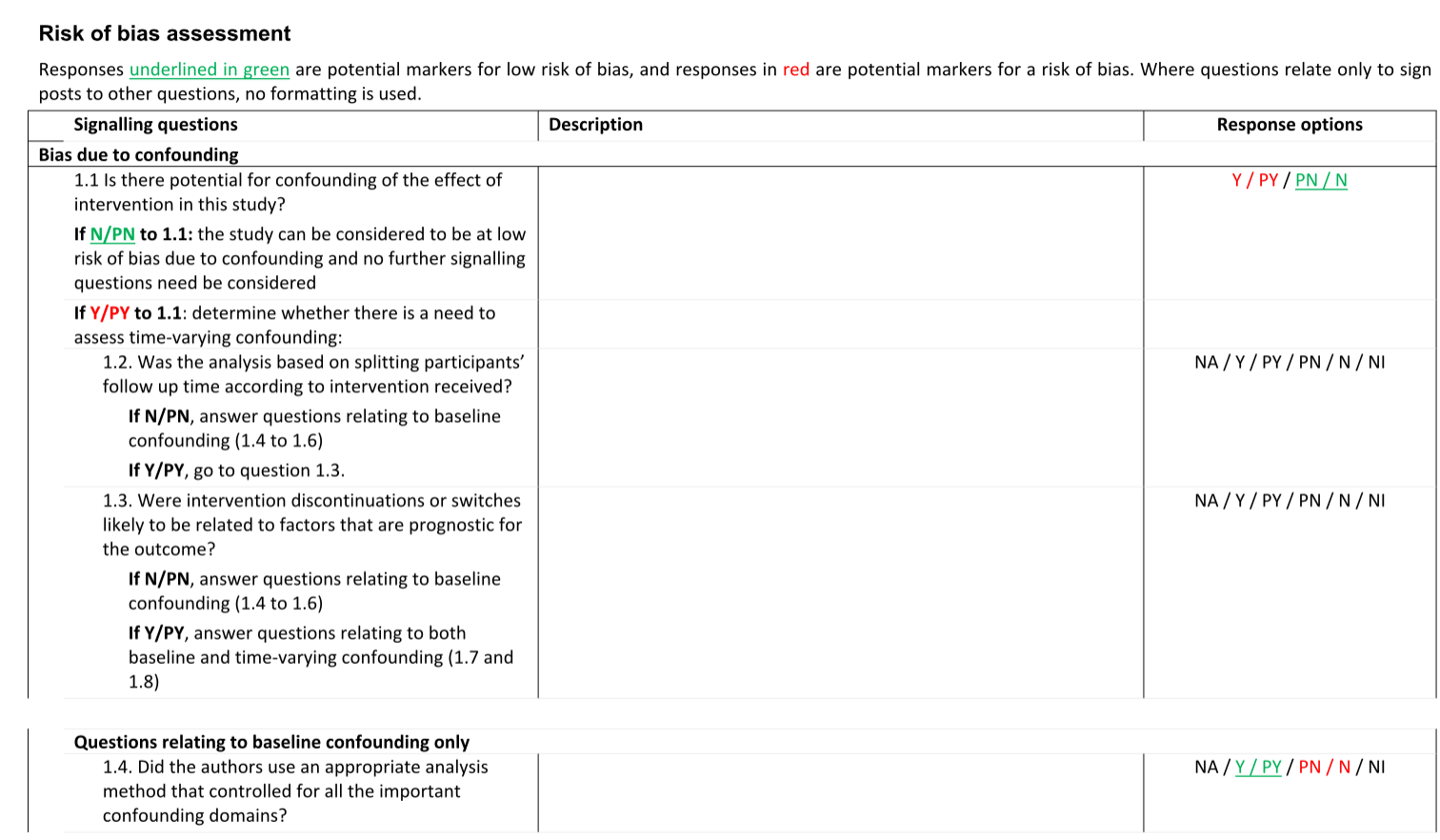


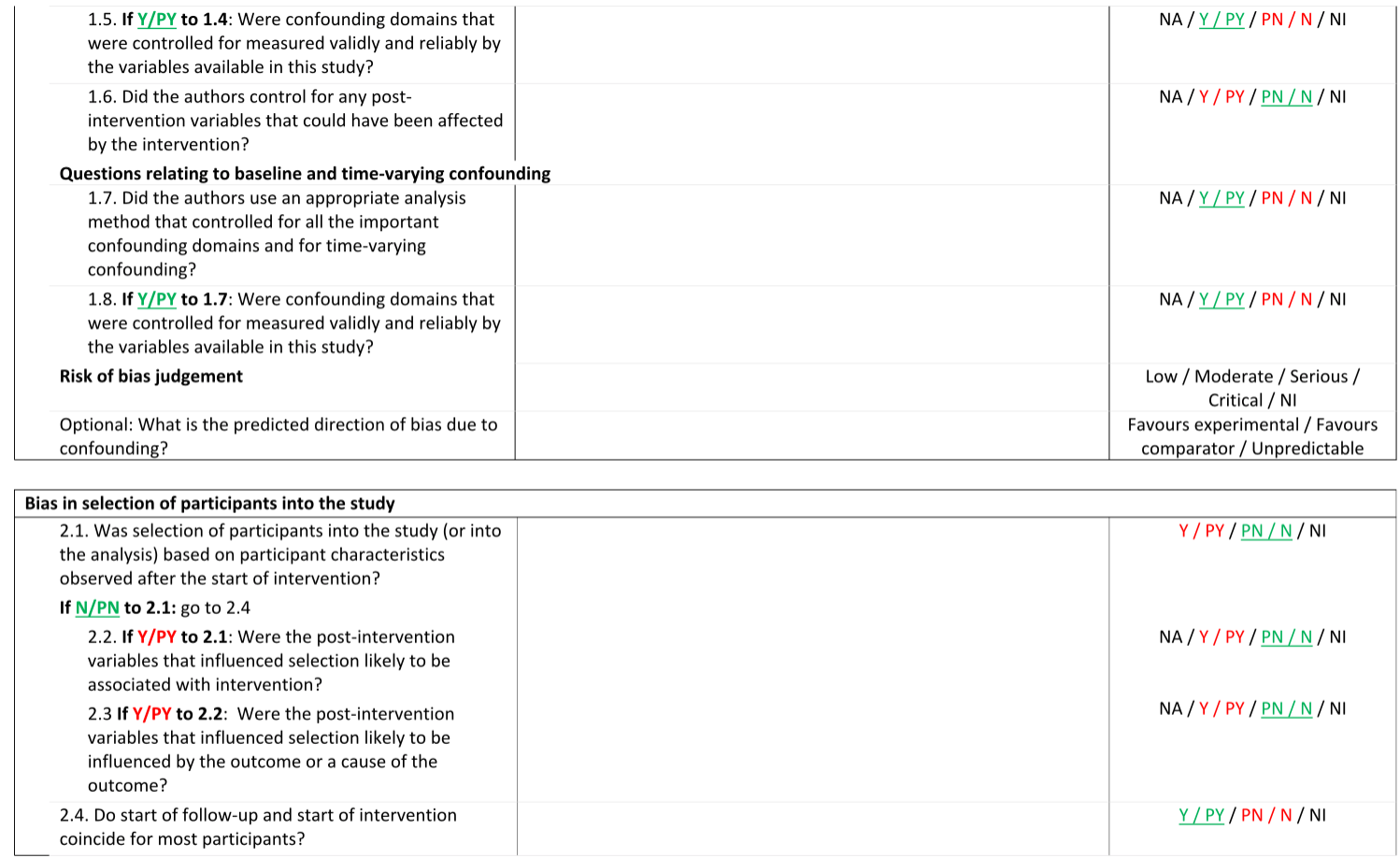


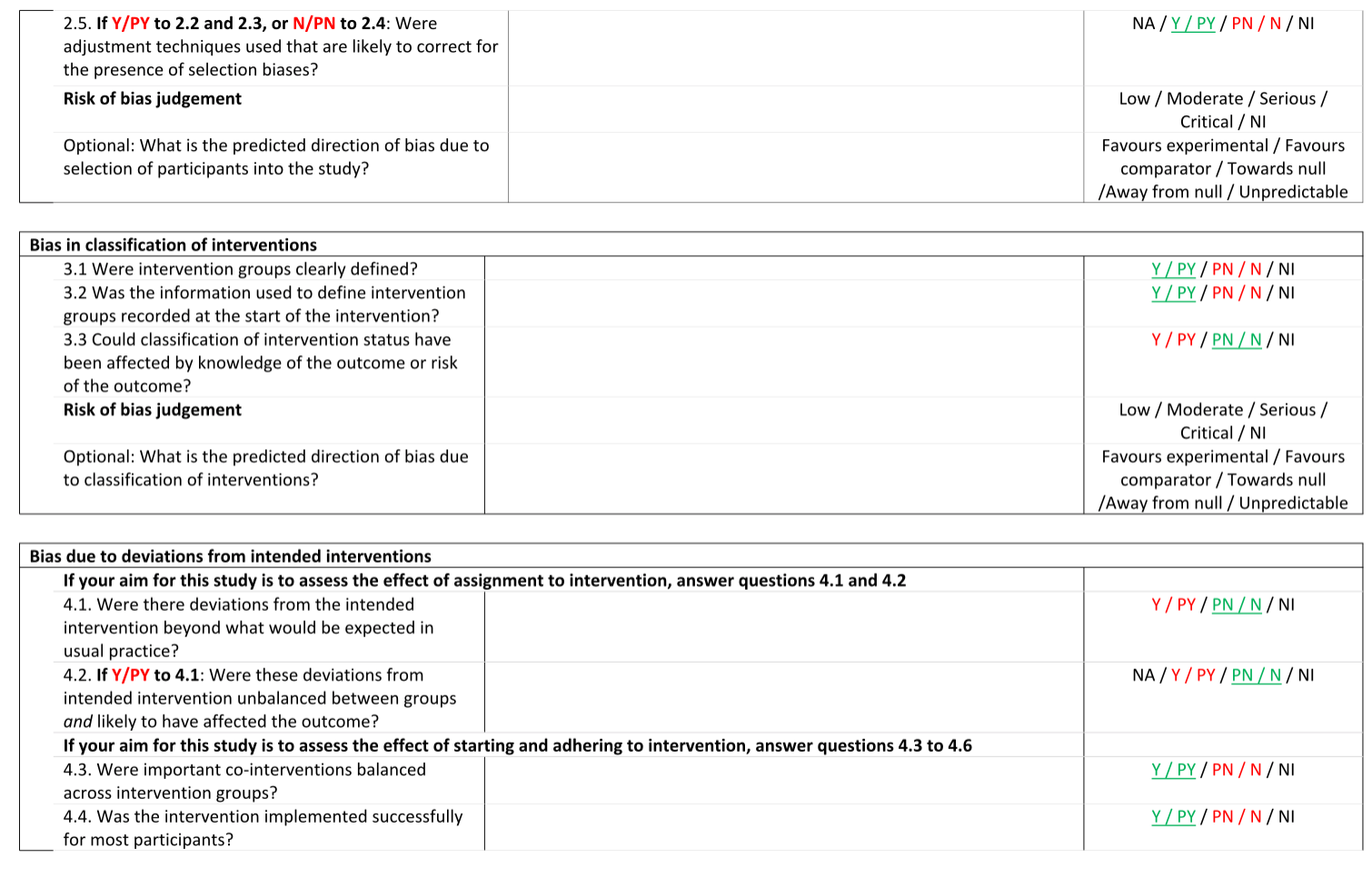


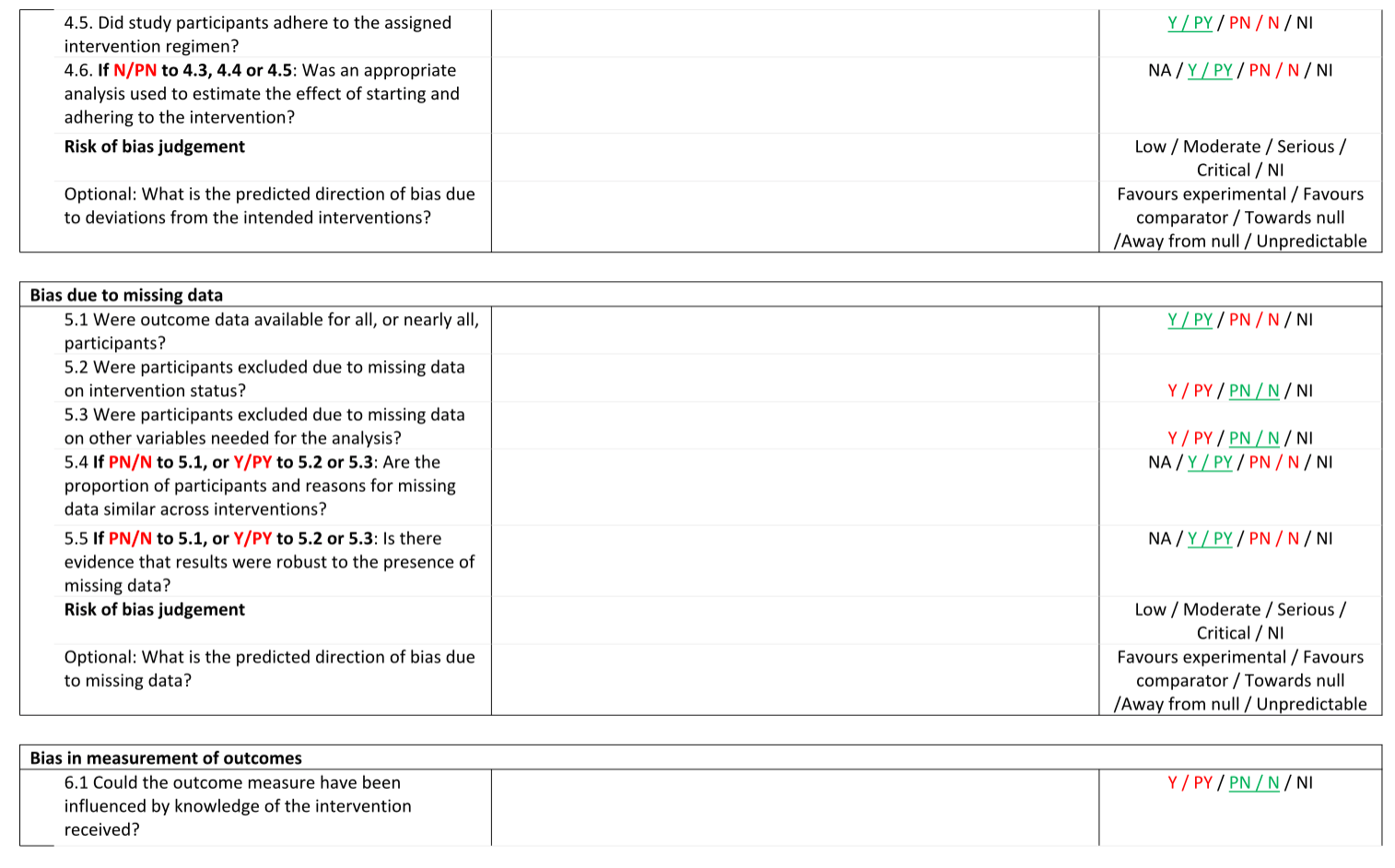

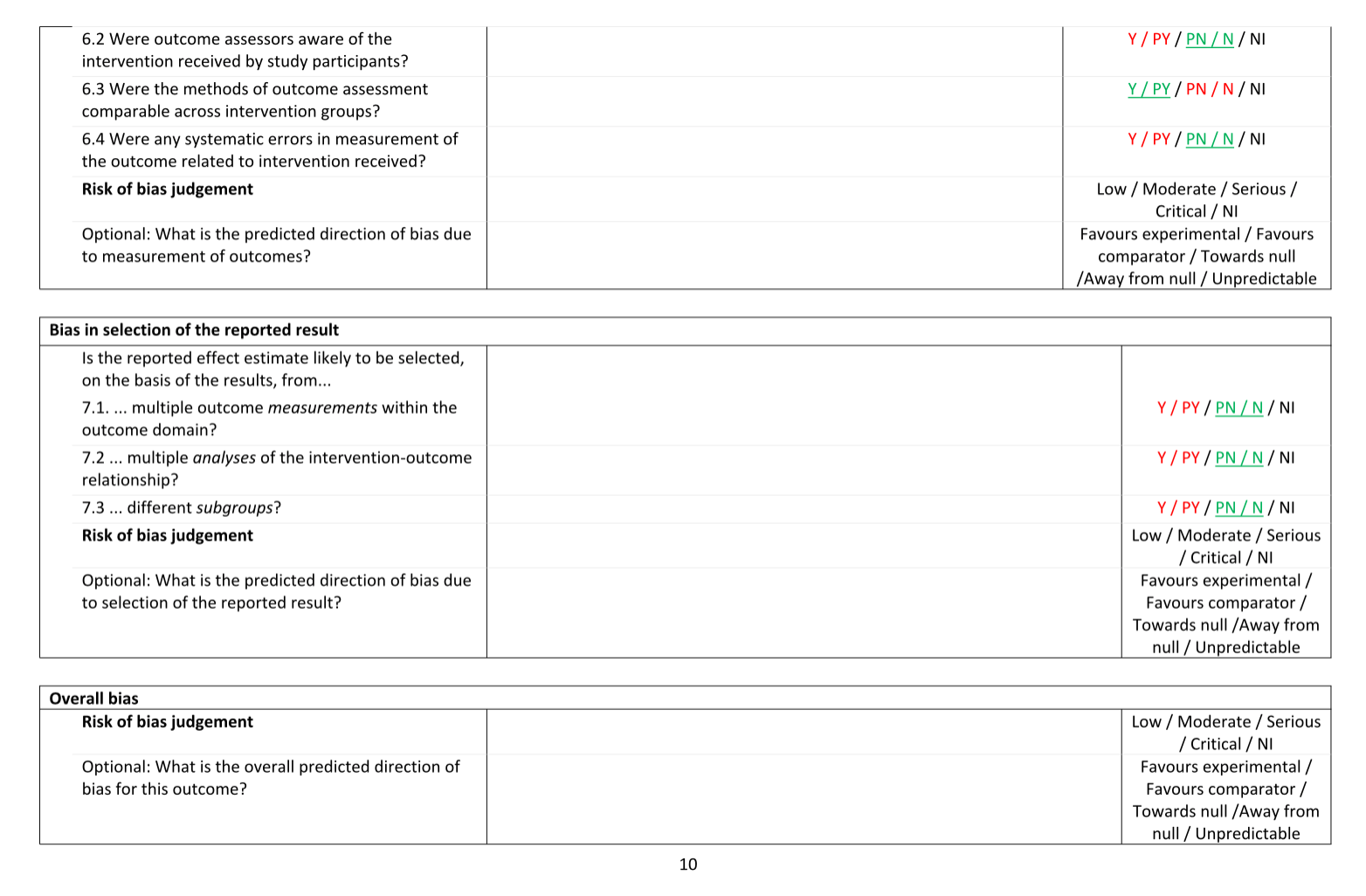


| **Supplementary Table 3: Characteristics of included studies ^[[1]](#footnote-1)^** | | | | | | |
| --- | --- | --- | --- | --- | --- | --- |
| **Study ID and Country** | **Design** | **Sample** | **Duration** | **Study purpose** | **Intervention** | **Outcomes** |
| **Aldana et al (2005) (45)**  *USA* | RCT | Baseline:  N= 145  6 weeks:  N= 141  6 months:  N= 137 | **Intervention:**  4 weeks  **Follow up:**  6 weeks  6 months | CVD risk reduction | - Coronary Health Improvement Project. - Educational course: lectures, textbooks, assignments, shopping tours and cooking demonstrations. - Encouraged to follow a plant based, unrefined diet which is low in fat, salt, sugar, cholesterol and animal protein. - Encouraged to walk for 30 minutes a day. - Control: no intervention. - **Multi-component: diet and physical activity** - **Type: educational** | Total energy intake (kcal/day), % calorie intake from fat, protein and carbohydrates, fruit and vegetable servings and fibre (g), wholegrain and meat servings, total dietary fat (g) (poly-, mono- and saturated fat), sodium (mg), total steps per week, BMI (kg/m², weight (kg), blood pressure, resting heart rate (bpm), glucose (mg/dl), cholesterol (mg/dL), CRP(mg/dL). |
| **Armitage (2015) (32)**  *UK* | Mixed measures | Baseline:  N=79  Follow up:  N= 62  (ITT: 79) | **Follow up:**  1 month | Increase fruit intake | - Implementation intentions. - Baseline: questionnaire with statement ‘we want you to plan to have an extra portion of fruit each day because forming plans has been shown to increase fruit intake’. - Control: given space to write plans. - Self-generated: standard implementation intention instructions. - Volitional help: tables with critical situations and responses - **Type: Behavioural** | Fruit intake (servings/day), metacognitive processing |
| **Armitage and Conner (2001) (52)**  *UK* | RCT | **Baseline:**  N=801  **Follow up:**  N=517 | **Follow up:**  5 months | Decrease fat intake | - Personalised feedback - All participants: measured total and saturated fat intake and given general information leaflets on UK recommendations for fat intake, morbidity and mortality levels from diet related conditions and covering letters. - Intervention: additional statement ‘currently you are deriving _% of calories from fat in your diet’. - **Type: Behavioural** | Total fat intake (g/day), Saturated fat intake (g/day). |
| **Barratt et al (1994) (56)**  *Australia* | RCT | Baseline: N=683  3 months:  N=417  6 months:  N=430 | **Follow up:**  3 months  6 months | Decrease serum cholesterol levels | - The Staff Healthy Heart Project - Baseline: Cholesterol screening and feedback - Self-help: educational workbook and monitoring sheet with suggested dietary changes - Nutrition course: (6 hours) Dietician led classes, workbooks, activities. - **Type: Educational** | Cholesterol (mmol/L), energy intake (MJ/day), total fat (% of energy intake, saturated fat (% of energy intake), fibre (g/mj) |
| **Blake et al**  **(2013) (35)**  *UK* | Multi-level ecological | Baseline:  N=1452  Follow up:  N=1134 | 5 years | Improve health and wellbeing | - Exercise classes/staff gym - Health campaigns - Community interventions - Health education and screening - Therapies (meditation, massage) - **Multi-component: diet and physical activity** - **Type: education, environmental** | Physical activity, mood, sleep, smoking, BMI (kg/m²), weight (KG), fruit and vegetable intake, water intake, sickness absence, job satisfaction, organisational commitment |
| **Brug et al (1999)**  **(55)**  *Netherlands* | RCT | Baseline:  N=347  Follow up:  N=315 | 4 weeks | Decrease fat intake  Increase fruit and vegetable intake | - Computer tailored nutritional education - Control: personalised dietary feedback (fat, fruit and vegetable intake). - Intervention: tailored letters plus feedback on personal outcome expectancies, perceived social influences and self-efficacy expectations. - **Type: educational** | BMI (kg/m²), fat score, servings of fruit, vegetables, intention to reduce fat, intention to increase fruit and vegetable intake, self-rated fat, fruit and vegetable intake. |
| **Choy et al (2017) (57)**  *Hong Kong* | RCT  *pilot* | N=42 | 8 weeks | Weight management | - Nutritional wellness programme - Baseline: 45-minute dietary education session - Intervention: tailored weight management (individual and telephone counselling, pamphlets, text messages) - Control: individual counselling, pamphlets - **Type: educational, behavioural** | Body weight (kg), BMI (kg/m²), serum fasting glucose (mg/dL), fasting cholesterol (mg/dL), triglyceride, high-density lipoprotein, low density lipoprotein, body fat %, body mass. |
| **Cockcroft et al (1994) (53)**  *UK* | RCT | Baseline:  N= 297  Follow up:  N=83 | Intervention:  1 week  Follow up:  6 months | Health promotion | - Baseline: health measurements - Control: given measurement results - Intervention: advice about how to change aspects of their health and goal setting. - **Multi component – diet and physical activity** - **Type: Educational, Behavioural** | Free time exercise, BMI (kg/m²), perception of health, diet score, alcohol (units/week), stress, forced expiratory volume |
| **Dawson et al (2006) (37)**  *Canada* | NR  *Pre-and post-intervention* | Baseline:  N=504  Follow up:  N=258 | NR | Increase availability of healthier food choices. | - Eat Smart! Workplace cafeteria program (nutrition component) - Questionnaires sent to staff members - **Type: environmental** | Frequency of cafeteria visits and purchases, program awareness, attitudes about the program, self- reported behaviour change. |
| **Doran et al (2018) (18)**  *USA* | RCT  *Cluster* | Baseline:  N=98  6 months:  N=59  9 months:  N=52  12 months:  N=45  (ITT=98) | Intervention: 9 months  Follow up:  6months  9 months  12 months | CVD risk reduction | - Worksite Heart Health Improvement Project - Environment and policy assessment - Group education - Motivation and active engagement - Technology enhanced motivation - Booster and long-term adherence - **Multi-component: diet, physical activity and stress** - **Type: educational, behavioural** | Mood, dietary salt and fat intake (g/week), sleep quality and duration |
| **Geaney et al (2011) (31)**  *Ireland* | Cross sectional | Baseline:  N=100  Follow up:  N=100 | Follow up:  3 years post- initiation | Examine the impact of a structured catering initiative on food choices | - Hospital cafeteria implemented a catering initiative to provide nutritious food while reducing sugar, fat and salt intakes. - Control cafeteria: no changes. - 24-hour dietary recall questionnaire. - **Type: environmental** | Energy intake (kcal/d), total sugars (g/d), sucrose and fructose (g/d), total and saturated fat (g/d), protein (g/d), carbohydrates (g/d), salt (g/d), Vitamin K, calcium, iron, B6, B12, C and D. |
| **Gomel et al (1993) (19)**  *Australia* | RCT  *Cluster* | Baseline:  N=431  Follow up:  3 months: N=405  6 months:  N=370  12 months:  N= 362 | Intervention:  12 months  Follow up:  3 months  6 months  12 months | Health promotion  CVD risk reduction | Intervention 1: health risk assessment  Intervention 2: risk factor education  Intervention 3: behavioural counselling  Intervention 4: behavioural counselling plus incentives.  **Type: Educational, behavioural** | BMI (kg/m²), % body fat, blood pressure (mmHg), serum cholesterol (mg/dL), smoking status, aerobic capacity. |
| **Hasson et al (2018), Polak et al (2015) (38,39)**  *Jerusalem* | NR  Pre-and post-test | Baseline:  N=104  Follow up:  N=104 | Intervention:  3 months  Follow up:  18 months | Health promotion | - Promoting Healthy Lifestyles - Interactive lectures and workshops on motivating behavioural change, balanced nutrition and physical activity. - Group exercise and pedometer   **Multi-component: diet and physical activity**  **Type: behavioural, educational** | Attitudes toward a healthy lifestyle, physical activity (sessions/week, walking/week), balanced diet (daily consumption of fruits and vegetables, milk products, grains, and protein rich foods). |
| **Hussain et al**  **(2018)**  **(36)**  *Malaysia* | Single-arm experimental | N=131 | Intervention:  3 months | Weight reduction | - Motivational educational workshop on healthy lifestyle and diet - Weight loss target of 10% - Dietician counselling: asked to avoid high calorie diet, reduce portion sizes and increase fruits and vegetables. - Supervised exercise training - **Multicomponent: diet and physical activity.** - **Type: behavioural, educational** | Weight (KG), (BMI kg/m²), body fat%, waist circumference (cm). |
| **Lahiri and Faghri (2012) (40)**  *USA* | NR | Baseline:  N=72 | Intervention:  16 weeks  Follow up:  28 weeks | Weight management | - Behavioural weight management program - Weight loss action plans: encouraged reflections on lifestyle, set goals, weight loss advice. - Week 3: tailored educational consultations with a weight loss goal. - Incentive: $10 per pound/ pound and a half lost. - **Type: behavioural, financial** | Weight (ibs), absenteeism, presenteeism |
| **Lassen et al (2014) (25)**  *Denmark* | Quasi-experimental | Baseline:  N=270  Follow up:  N= 270 | Intervention:  6 weeks  Follow up:  6 months | Evaluate the effectiveness of serving healthy labelled meals to improve the nutritional quality of meals eaten in a worksite canteen. | - Intervention canteen: completing the Keyhole certification process where 50% of meals aimed to have healthy labels. - Control canteen: no changes. - Photo-weighing stations to measure intake before and after eating. - **Type: environmental** | Energy/meal (MJ), energy density, Fat (% of energy intake), fruit and vegetable (g/100g), Salt (g/100g), refined sugars (g/100g), wholegrain (g/100g). |
| **Leedo et al (2017) (24)**  *Denmark* | RCT  *crossover* | Baseline:  N=60  Follow up:  N=59 | Intervention:8 weeks | Examine the effect of increased availability of healthy meals and water at work | - Intervention period (4 weeks): Received a key-hole labelled meal, snack and bottled water during each shift. - Control period (4 weeks): instructed to continue with their habitual dietary intake. - **Multiple: reaction time/mood** - **Type: environmental** | Reaction time (go/no-go test), mood related scores, dietary intake (energy intake (kj/d), fat and saturated fat (% of energy intake), polyunsaturated fat (g), protein and carbohydrates (% of energy intake), dietary fibre (g/d), water (ml/d). |
| **Lemon et al (2010)**  **(46)**  *USA* | RCT  *Pair matched cluster* | Baseline:  N=806  12 months:  N=731  24 months:  N=648 | Intervention: 2 years  Follow up:  12, 24 months | Weight gain prevention | - Step Ahead: A worksite obesity prevention trial. - Control: no intervention - Intervention: social marketing campaign, environmental changes to promote physical activity and healthy eating, promotion of interpersonal support, workshops, newsletters. - **Type: environmental** - **Multi-component: diet and physical activity** | BMI (kg/m²), perceptions of organisational commitment to employee health, normative co-worker behaviours. |
| **Lowe et al (2010)**  **(47)**  *USA* | RCT | Baseline:  N=96  6 months:  N=77  12 months:  N=63 | Intervention:  3 months  Follow up: 6, 12 months | Improve macronutrient intake | - Control: environmental change (introduction of ten new low-energy density foods and food labels in the canteen. - Intervention: environmental change plus pricing incentives for purchasing low energy density foods. Education sessions about low energy density eating. - **Type: environmental, educational, financial** | Purchased kcal, % of calories from fat, protein and carbohydrates, blood lipid levels (total, HDL, LDL and TAG). |
| **Lusczynska and Haynes (2009)**  **(54)**  *UK* | RCT | Baseline:  N=182  Follow up:  (ITT: 182) | Intervention: 9 weeks  Follow up: 4 months | Increase fruit and vegetable intake and physical activity. | - Control: visual education materials referring to healthy nutrition and exercise. - Intervention: shortened versioned of educational materials plus planning forms. Asked to make plans to increase fruit and vegetable intake and physical activity. - **Multi-component: diet and physical activity** - **Type: educational, behavioural** | Fruit and vegetable intake (servings/day), physical activity, BMI (kg/m²), intentions to increase fruit and vegetable intake and physical activity, self-efficacy. |
| **Milich et al (1976) (41)**  *USA* | NR | Baseline:  N=450  Follow up:  N=450 | Intervention:  4 weeks | Test the effects of visual presentation of caloric values on purchases. | - Every item of food was labelled with its name and caloric value - Food purchases recorded by two judges; also classified customers as ‘normal’, ‘overweight’ or ‘obese’. - **Type: environmental** | Calories purchased; money spent. |
| **Nepper et al (2020) (26)**  *USA* | Quasi-experimental | Baseline:  N=41  Follow up:  N=41 | Intervention:  16 weeks  Follow up:  3, 6 months | Weight loss, improve physical, behavioural and mental wellbeing. | - Better Living programme - Weekly group meetings to improve nutrition and physical activity, promote cognitive and behavioural changes and improve mental health and sleep quality. - **Multi-component: diet, physical activity and mental health.** - **Type: educational** | Weight (KG), BMI (kg/m²), blood pressure (mmHg), eating >5 servings of fruit and vegetables, consuming high fat foods, exercise (30 minutes/d), sleep duration and quality, depression. |
| **Racette et al (2009) (23)**  *USA* | RCT  *cohort* | Baseline:  N=151  Follow up:  N=123 | Intervention and follow up:  12 months | Health Promotion  CVD risk reduction | - Control: assessment only. Personal health report - Intervention: assessment and intervention. Promotion of physical activity and favourable dietary patterns using pedometers, healthy snack cart, weight watchers’ meetings, exercise classes, seminars, team competitions and rewards. - **Multi-component: diet and physical activity** - **Type: environmental, behavioural** | BMI (kg/m²), weight (kg), %/kg fat mass, fat free mass (kg), blood pressure, fitness, heart rate (bpm), lipids (total cholesterol, HDL, LDL, TAG), fasting glucose (mg/dL) and Framingham coronary heart disease risk. |
| **Rigsby et al (2009) (42)**  *USA* | NR | Baseline:  N=77  Follow up:  N=72 | 8 weeks | Weight loss with peer support and healthy competition | - Weight loss competition - Participated as part of a group or as individuals - No specific intervention - **Type: behavioural, financial** | Weight (ib), BMI, body fat (%) |
| **Ross and Wing (2016) (43)**  *USA* | NR | Baseline:  N=75  3 months:  N=70  6months:  N=67  (ITT: 75) | Intervention:  12 weeks  Follow up:  6 months | Weight loss | - Initial in-person group education session on weight management, prescribed calorie, fat and physical activity goals. - Internet weight loss program: weekly education - Optional in-person counselling if weight loss ineffective at four weeks. - Financial incentives. - **Multi-component: diet and physical activity** - **Type: educational, financial** | Weight (KG), minutes of physical activity. |
| **Scapellato et al (2018) (44)**  *Italy* | NR | Baseline:  318  6 months:  167  12 months:  NR | Intervention and follow up:  12 months | Health promotion  CVD risk reduction | - Customised advice on eating habits and level of physical activity - Motivational counselling to identify and set goals for their physical activity and nutrition - 3 months: motivational support via phone counselling - **Multicomponent: diet and physical activity** - **Type: behavioural** | Physical activity (type, frequency, duration), blood pressure (mmHg), waist circumference (cm), BMI (kg/m²), cholesterol (total, LDL, HDL, TAG) (mg/dL), blood glucose (mg/dL). |
| **Sorensen (20) (21) and Hunt et al (2001) (22)**  *USA* | RCT  *Cluster* | Baseline:  N= 1359  Follow up:  N= NR | Intervention:  Follow up:  19.5 months | Increase fruit and vegetable consumption | - Control: minimal intervention (offered to all, national 5-a-day campaign,5-a-day presentation and taste test) (8 sites). - Worksite intervention: advisory boards, media campaign, presentations, videos, group sessions, individual advice and environmental change (increase in fruit and vegetables in vending machines, taste-tests and point-of-choice labelling of fruit and vegetables) (7 sites) - Worksite plus family intervention: family-focused interventions in the worksite programme, learn-at-home programme, newsletter, family festival and mailings (7 sites). - **Type: Environmental, educational, behavioural** | Fruit and vegetable intake (servings/day) |
| **Stites et al (2015) (48)**  *USA* | RCT  *Prospective cohort* | Baseline:  N=26  Follow up:  N=26 | Intervention:  8 weeks | Promote healthier lunch purchases | - Baseline (4 weeks): - Intervention (4 weeks): mindful eating training, pre ordered lunches, price discounts. - Reduced intervention (4 weeks): pre ordered lunches without price discounts. - **Type: behavioural, financial** | Kcal and fat grams purchased. |
| **Tate, Wing and Winett (2001) (49)**  *USA* | RCT | Baseline:  N=91  Follow up:  N=65 | Intervention:  6 months | Weight loss | - Control: internet education. One face-to-face group weight loss session and access to a web site with links to weight loss resources. - Intervention: same as controls plus internet behaviour therapy. Additional twenty-four weekly behavioural lessons via email, self-monitoring diaries and individualised therapist feedback. - **Type: behavioural, educational** | Weight (kg), waist circumference (cm). |
| **Thorndike et al (2014/2012), Levy et al (2012), Dashti et al (2020) (27-30)**  *USA* | Longitudinal | Baseline and follow up:  N=2285 | Intervention and follow up: 24 months | Promote healthier food choices | - Traffic-light labelling: cafeteria items labelled green (healthy), yellow (less healthy) or red (unhealthy) - Choice architecture: items rearranged to make healthy items more accessible. - **Type: environmental** | Cafeteria sales of green or red items. |
| **Torquati et al (2018) (34)**  *Australia* | Pilot intervention (pre- and post-test) | Baseline:  N=47  3 months:  N=27  6 months:  N=12 | Intervention: 3 months  Follow up:  6 months | Promote a healthy diet and physical activity | - Diet quality: limiting availability of sweets/chocolate, bringing healthy meals to work - Physical activity: colleague support and motivation, active transport, pedometers, exercise before and after shifts - **Multi-component: diet and physical activity** - **Type: environmental, behavioural** | Physical activity behaviour, diet behaviour (Australian food score), energy intake (kJ/d), fruit and vegetables (%), discretionary food (% energy), weight, BMI(kg/m²), waist circumference (cm), blood pressure (mmHg), self-rated health, diet and physical activity self-efficacy and social support. |
| **Townsend et al (2016) (50)**  *USA* | NR | Baseline and follow up:  Health centres: N=64  Health systems: N=31 | Intervention: 3 months | Reduce obesity disparities in Native Hawaiians. | - Diabetes Prevention Program Lifestyle Intervention (adapted) - Part of a larger 9-month weight loss maintenance intervention (PILI lifestyle program). - Eight lessons on eating healthy, increasing physical activity, motivational techniques, problem solving.   **Multi component: diet and physical activity**  **Type: educational** | Body weight (KG), blood pressure (mmHg), physical functioning (walk test), physical activity (frequency), fat intake (diet score), exercise and eating self-efficacy, family and community support. |
| **Van Kleef et al (2012) (33)**  *Netherlands* | Mixed – two-factor experimental | N=158 | 4 weeks | Examine the effects and interplay between shelf arrangement and assortment structure on consumer choices. | - Laboratory: assigned to one of four conditions. Shown a picture of a shelf display with snacks. Manipulated amount and position of healthy and unhealthy snacks. Asked to choose a snack. - Field: snacks displayed in a hospital canteen. Snack arrangement changed weekly for four weeks. - **Type: environmental** | Snack choice |
| **Winick et al (2002) (51)**  *USA* | Longitudinal study | Baseline:  492  Follow up:  393 | c  Intervention: 12 weeks | Weight reduction at high stress worksites. | - Two meal replacements daily - Received package with 14 portion-controlled servings each week with 12 nutritious snack bars. - Third ‘sensible’ meal of choice (up to 500 calories). - **Type: behavioural** | Weight (KG), BMI (kg/m²) |

**
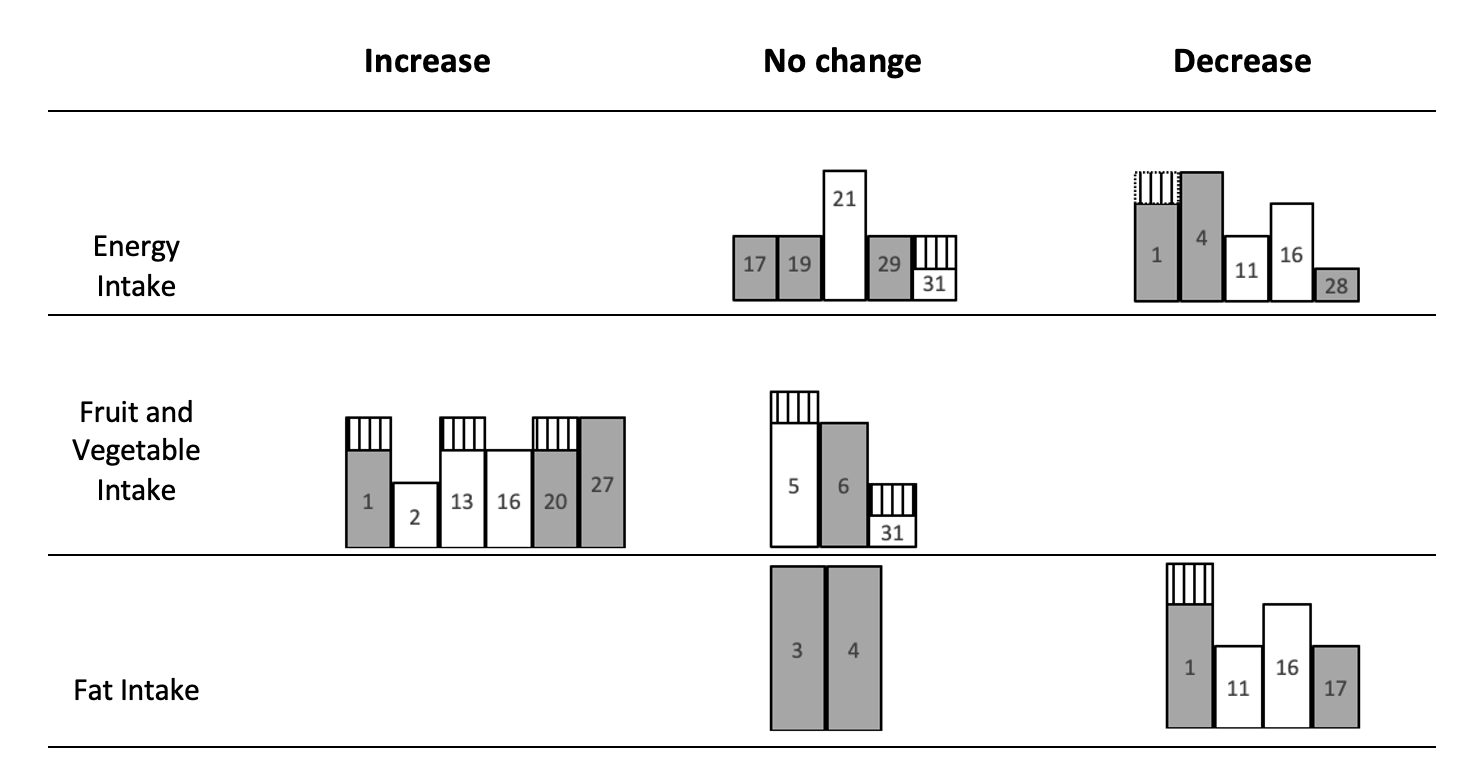
Supplemental Figure 1: Harvest plot for selected dietary outcomes.**

Harvest plot displaying direction of effect and study quality measures for energy, fruit, vegetable and fat intake. 50% of interventions produces significant decreases in energy intake while the remaining 50% did not produce a significant change. No clear trends were observed in terms of sample size, study design, or use of physical activity measures. Fruit and vegetable intake increased in 6/9 interventions. 5/6 of these interventions involved a minimum of 100 participants, 50% were RCTs and 50% used physical activity measures. No trends were observed in studies reporting no change in terms of sample size, study design and physical activity. Fat intake increased in four interventions and did not significantly change in two interventions. No clear trends were observed in terms of sample size, study design (50% RCTs) or use of physical activity measures in those reporting significant decreases. The two studies reporting no effect involved 6-800 participants, were both RCTs and neither used physical activity measures.

**Supplemental Figure 2: Harvest Plot for selected health outcomes.**


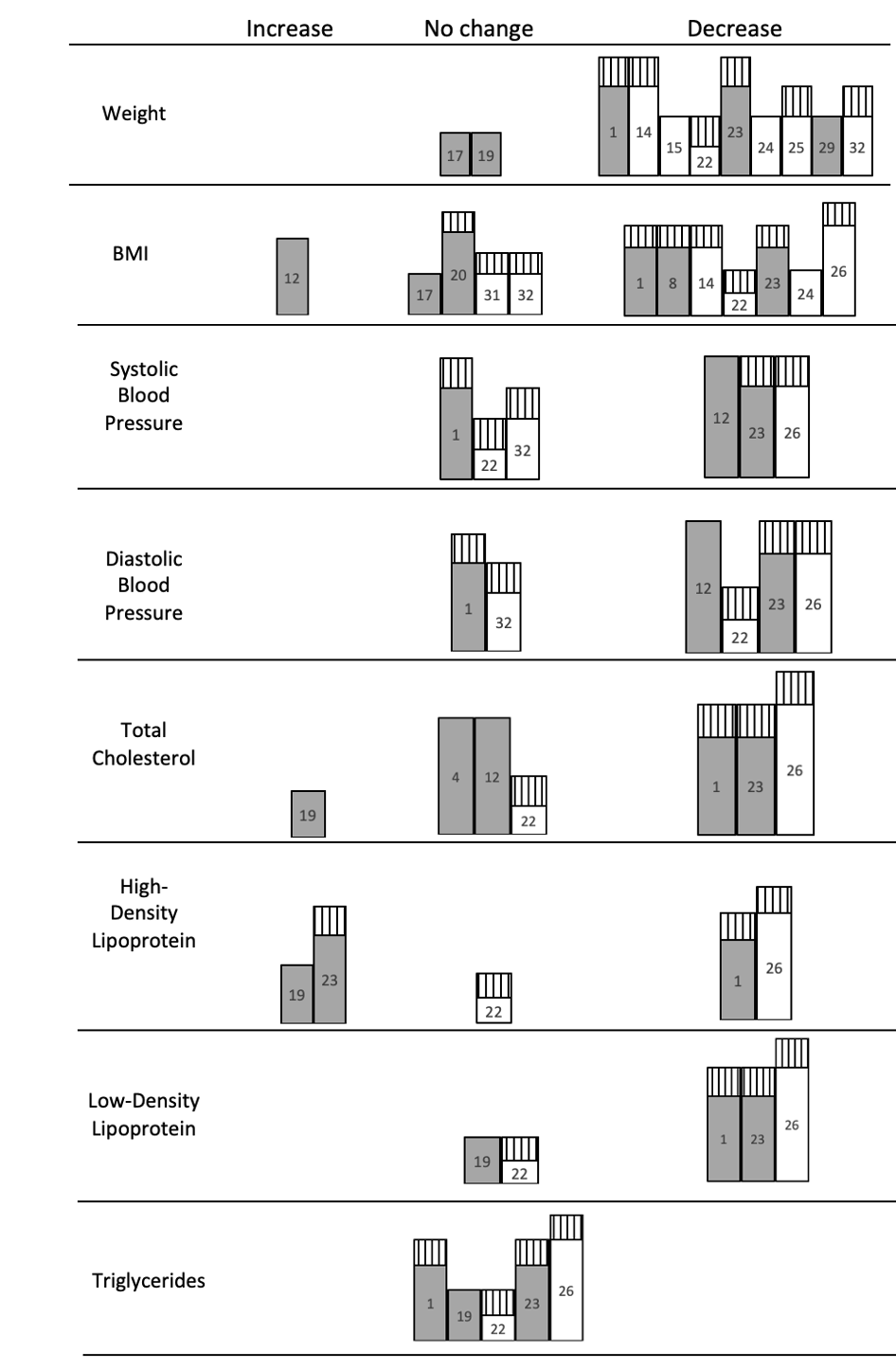


Harvest plot displaying direction of effect and study quality measures for weight, BMI, blood pressure and serum cholesterol levels. Weight significantly decreased in nine interventions and no significant change was observed in two. 8/9 interventions reporting a significant decrease involved at least 70 participants, 2/3 were NRCTs and 2/3 of interventions used physical activity measures. The two interventions reporting no effect involved 60-100 participants, were RCTs and did not use any physical activity measures. BMI significantly decreased in seven interventions, significantly increased in one intervention and no significant change was observed in four. Considerable variation was observed in sample size and study design in interventions reporting significant decreases, however the majority utilised physical activity measures. Amongst those displaying no effect, sample size and study design also varied, with 75% utilising physical activity measures. The RCT reporting a significant increase in BMI had a large sample size. Systolic blood pressure significantly increased in 50% of interventions and the remaining 50% did not produce significant change. Diastolic blood pressure significantly reduced in four interventions and produced no significant change in two. Studies reporting significant decreases in systolic and diastolic blood pressure involved at least 100 participants; 2/3 were RCTs and 2/3 used physical activity measures. A further study reported a significant decrease in diastolic blood pressure but not systolic; this study had a sample size of 41, was a NRCT and used physical activity measures. Studies reporting no effect displayed no similarities in sample size or study design, but both used physical activity measures. No clear direction of effect was observed in serum cholesterol levels. Studies reporting significant decreased in total and LDL cholesterol all implemented physical activity measures and involved 145+ participants. No trends were observed in study design for any cholesterol measurements. No interventions produced significant changes in TAG levels.

**Supplemental Figure 3: Harvest plot key for Figures 2 and 3 detailing study quality^[[2]](#footnote-2)^**


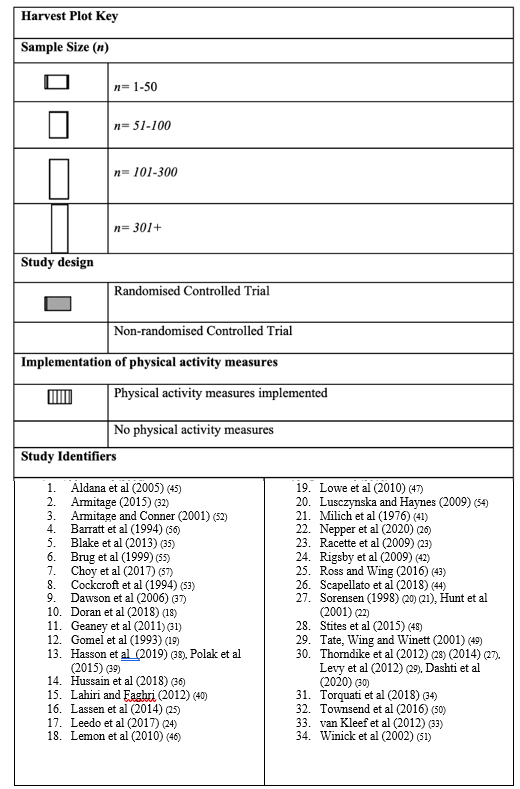


1. BMI – body mass index; CRP – C-reactive protein; CVD – cardiovascular disease; HDL – high-density lipoprotein; LDL – low-density lipoprotein; NR – not reported; RCT – Randomised controlled trial; TAG- triglycerides. [↑](#footnote-ref-1)
2. Bar height represents sample size; lining represents use of physical activity measures;

   shading represents RCTs [↑](#footnote-ref-2)
